# Supplementary material for: The identification and treatment of mental health and substance misuse problems in sexual assault services: A systematic review
Source: PLoS One. 2020 Apr 10;15(4):e0231260. doi: 10.1371/journal.pone.0231260 (PMC7147790; doi:10.1371/journal.pone.0231260)
Supplement: S2 File — (DOCX) [file pone.0231260.s002.docx]

**Additional File 2_Search terms**

**Records identified through database searching (n = 3,774)**

1. **PsychInfo (1975 to August week 2 2018), 1,093 hits**
2. "rape suite*".mp.
3. "sexual assault referral team*".mp.
4. "sexual assault nurse examiner*".mp.
5. ("sexual assault service program*" or "sexual assault service programme*").mp.
6. ("sexual assault domestic violence care” adj2 “treatment centre*" or "sexual assault domestic violence care adj2 treatment center*").mp.
7. ("child sexual assault" adj5 (service* or team* or center* or centre*)).mp.
8. (“child sexual abuse” adj5 (service* or team* or center* or centre*)).mp.
9. "sexual assault resource*".mp.
10. "specialist sexual violence service*".mp.
11. ("independent rape centre*" or "independent rape center*").mp.
12. ("independent sexual assault center*" or "independent sexual assault centre*").mp.
13. ("thuthuzela care centre*" or "thuthuzela care center*").mp.
14. ("sexual assault referral center*" or "sexual assault referral centre*").mp.
15. SARC.mp.
16. SARCs.mp.
17. SVSs.mp.
18. "SART-SANE".mp.
19. SART
20. SARTs.mp.
21. SASPs.mp.
22. NASASV.mp.
23. NASASVs.mp.
24. SSVS.mp.
25. SSVSs.mp.
26. (“sexual assault” adj5 (team* or center* or centre* or service*)).mp.
27. (“sexual abuse” adj5 (team* or center* or centre* or service*)).mp.
28. (“sexual violence” adj5 (team* or center* or centre* or service*)).mp.
29. (“sexual assault response” adj5 (team* or center* or centre* or service*)).mp.
30. (“rape center*” or “rape centre*”).mp.
31. (“rape victim” adj5 (centre* or center* or team* or service*)).mp.
32. ("nordic model" adj5 (rape* or sexual assault or sexual violence or sexual abuse)).mp.
33. (“one-stop-shop” adj5 (rape or sexual assault or sexual violence or sexual abuse)).mp.
34. ("one stop shop" adj5 (rape or sexual assault or sexual violence or sexual abuse)).mp.
35. (association adj3 "service* against sexual violence").mp.
36. ("nordic model" adj5 "rape victim center*").mp.
37. ("nordic model" adj5 "rape victim centre*").mp.
38. **Ovid MEDLINE(R) and Epub Ahead of Print, In-Process & Other Non-Indexed Citations, Daily, and Versions(R), 1975 to August 16, 2018, 1,026 hits**
39. "rape suite*".mp.
40. "sexual assault referral team*".mp.
41. "sexual assault nurse examiner*".mp.
42. ("sexual assault service program*" or "sexual assault service programme*").mp.
43. ("sexual assault domestic violence care adj2 treatment centre*" or "sexual assault domestic violence care adj2 treatment center*").mp.
44. ("child sexual assault" adj3 (service* or team* or center* or centre*)).mp.
45. (“child sexual abuse” adj5 (service* or team* or center* or centre*)).mp.
46. "sexual assault resource*".mp.
47. "specialist sexual violence service*".mp.
48. ("independent rape centre*" or "independent rape center*").mp.
49. ("independent sexual assault center*" or "independent sexual assault centre*").mp.
50. ("thuthuzela care centre*" or "thuthuzela care center*").mp.
51. ("sexual assault referral center*" or "sexual assault referral centre*").mp.
52. SARC.mp.
53. SARCs.mp.
54. SVSs.mp.
55. "SART-SANE".mp.
56. SARTs.mp.
57. SASPs.mp.
58. NASASV.mp.
59. NASASVs.mp.
60. SSVS.mp.
61. SSVSs.mp.
62. (“sexual assault” adj5 (team* or center* or centre* or service*)).mp.
63. (“sexual abuse” adj5 (team* or center* or centre* or service*)).mp.
64. (“sexual violence” adj5 (team* or center* or centre* or service*)).mp.
65. (“sexual assault response” adj5 (team* or center* or centre* or service*)).mp.
66. (“rape center*” or “rape centre*”).mp.
67. (“rape victim” adj5 (centre* or center* or team* or service*)).mp.
68. ("nordic model" adj5 (rape or sexual assault or sexual violence or sexual abuse)).mp.
69. (“one-stop-shop” adj5 (rape or sexual assault or sexual violence or sexual abuse)).mp.
70. ("one stop shop" adj5 (rape or sexual assault or sexual violence or sexual abuse)).mp.
71. (association adj3 "service* against sexual violence").mp.
72. ("nordic model" adj5 "rape victim center*").mp.
73. ("nordic model" adj5 "rape victim centre*").mp.
74. **CINAHL Plus, search ran on 17/08/2018 (Limiters: Publication Year: 1975-2019; Exclude MEDLINE records; Human), 633 hits**

"rape suite*" or "sexual assault referral team*" or "sexual assault nurse examiner*" or "sexual assault service program*" or "sexual assault service programme*" or "sexual assault domestic violence care" N2 "treatment centre*" or "sexual assault domestic violence care" N2 "treatment center*" or "child sexual assault" N4 (service* or team* or center* or centre*) or “child sexual abuse” N4 (service* or team* or center* or centre*) or "sexual assault resource*" or "specialist sexual violence service*" or "independent rape centre*" or "independent rape center*" or "independent sexual assault center*" or "independent sexual assault centre*" or "thuthuzela care centre*" or "thuthuzela care center*" or "sexual assault referral center*" or "sexual assault referral centre*" or SARC or SARCs or SAC or SACs or SVS or SVSs or "SART-SANE" or SART or SARTs or SASP or SASPs or NASASV or NASASVs or SSVS or SSVSs or TCC or TCCs or “sexual assault” N4 (team* or center* or centre* or service*) or “sexual abuse” N4 (team* or center* or centre* or service*) or “sexual violence” N4 (team* or center* or centre* or service*) or “sexual assault response” N4 (team* or center* or centre* or service*) or "rape center*" or "rape centre*" or "rape victim" N4 (centre* or center* or team* or service*) or "nordic model" N4 (rape* or sexual assault or sexual violence or sexual abuse) or “one-stop-shop” N4 (rape or sexual assault or sexual violence or sexual abuse) or "one stop shop" N4 (rape or sexual assault or sexual violence or sexual abuse) or association N2 "service* against sexual violence" or "nordic model" N4 "rape victim center*" or "nordic model" N4 "rape victim centre*"

1. **IBSS, 1975 to 17/08/2018, 1,022 hits**

("rape suite*" OR "sexual assault referral team*" OR "sexual assault nurse examiner*" OR "sexual assault service program*" OR "sexual assault service programme*" OR "sexual assault domestic violence care" NEAR/2 "treatment centre*" OR "sexual assault domestic violence care" NEAR/2 "treatment center*" OR "child sexual assault" NEAR/4 service* OR "child sexual assault" NEAR/4 team* OR "child sexual assault" NEAR/4 center* OR "child sexual assault" NEAR/4 centre* OR "child sexual abuse" NEAR/4 service* OR "child sexual abuse" NEAR/4 team* OR "child sexual abuse" NEAR/4 center* OR "child sexual abuse" NEAR/4 centre* OR "sexual assault resource*" OR "specialist sexual violence service*" OR "independent rape centre*" OR "independent rape center*" OR "independent sexual assault center*" OR "independent sexual assault centre*" OR "thuthuzela care centre*" OR "thuthuzela care center*" OR "sexual assault referral center*" OR "sexual assault referral centre*" OR SARC OR SARCs OR SVS OR SVSs OR "SART-SANE" OR SART OR SARTs OR SASP OR SASPs OR NASASV OR NASASVs OR SSVS OR SSVSs OR "sexual assault" NEAR/4 team* OR "sexual assault" NEAR/4 center* OR "sexual assault" NEAR/4 centre* OR "sexual assault" NEAR/4 service* OR "sexual abuse" NEAR/4 team* OR "sexual abuse" NEAR/4 center* OR "sexual abuse" NEAR/4 centre* OR "sexual abuse" NEAR/4 service* OR "sexual assault response" NEAR/4 team* OR "sexual assault response" NEAR/4 center* OR "sexual assault response" NEAR/4 centre* OR "sexual assault response" NEAR/4 service* OR "Nordic model" NEAR/4 rape* OR "Nordic model" NEAR/4 "sexual assault" OR "Nordic model" NEAR/4 "sexual abuse" OR "Nordic model" NEAR/4 "sexual violence" OR "one stop shop" NEAR/4 rape OR "one stop shop" NEAR/4 "sexual assault" OR "one stop shop" NEAR/4 "sexual abuse" OR "one stop shop" NEAR/4 "sexual violence" OR "one-stop -shop" NEAR/4 rape OR "one-stop-shop" NEAR/4 "sexual assault" OR "one-stop-shop" NEAR/4 "sexual abuse" OR "one-stop-shop" NEAR/4 "sexual violence" OR association NEAR/2 "service* against sexual violence" OR "Nordic model" NEAR/4 "rape victim centre*" OR "nordic model" NEAR/4 "rape victim center*") NOT (rat or rats or pigeon or pigeons or monkey or monkeys)
