# Supplementary material for: The identification and treatment of mental health and substance misuse problems in sexual assault services: A systematic review
Source: PLoS One. 2020 Apr 10;15(4):e0231260. doi: 10.1371/journal.pone.0231260 (PMC7147790; doi:10.1371/journal.pone.0231260)
Supplement: S3 File — (DOCX) [file pone.0231260.s003.docx]

**Additional file 3_MMAT scores**

**Table 1: MMAT scores for evaluation studies**

| **Category of study designs** | **Methodological quality criteria** | Acierno (2003) | Miller (2015) | Nixon (2016) | Resnick (2007) | Rheingold (2013) | Walsh (2017) |
| --- | --- | --- | --- | --- | --- | --- | --- |
| **Screening questions (for all types)** | S1. Are there clear research questions? | Y | Y | Y | Y | Y | Y |
|  | S2. Do the collected data allow to address the research questions? | Y | Y | Y | Y | Y | Y |
| **2. Randomised controlled trials** | 2.1. Is randomization appropriately performed? | c/t | c/t | N | N | Y | Y |
|  | 2.2 Are the groups comparable at baseline? | Y | Y | Y | Y | Y | Y |
|  | 2.3. Are there complete outcome data? | N | N | N | N | N | N |
|  | 2.4. Are outcome assessors blinded to the intervention provided? | Y | Y | Y | Y | N | Y |
|  | 2.5 Did the participants adhere to the assigned intervention? | Y | Y | Y | Y | Y | Y |
|  | **TOTALS** | 3 | 3 | 3 | 3 | 3 | 4 |

Note: c/t=can’t tell

**Table 2: MMAT scores for stakeholders’ views studies**

| **Category of study designs** | **Methodological quality criteria** | Ahrens (2000) | Belew (2012) | Bows (2018) | Brooker (2015) | Brooker (2018) | Burton (2002) | Campbell (1998) | Campbell (2005) | Campbell (2006) | Campbell (2013) | Clark (1998) | Cole (2007) | Cole (2008) | COSAI (2012) | Cowley (2014) | Downing (2012) | Du Mont (2004) | Du Mont (2009) |
| --- | --- | --- | --- | --- | --- | --- | --- | --- | --- | --- | --- | --- | --- | --- | --- | --- | --- | --- | --- |
| **Screening questions (for all types)** | S1. Are there clear research questions? | Y | Y | Y | Y | Y | Y | Y | Y | Y | Y | Y | Y | Y | Y | Y | Y | Y | Y |
|  | S2. Do the collected data allow to address the research questions? | Y | Y | Y | Y | Y | Y | Y | Y | Y | Y | Y | Y | Y | Y | Y | Y | Y | Y |
| **1. Qualitative** | 1.1 Is the qualitative approach appropriate to answer the research question? | Y |  | Y | c/t |  |  | Y |  |  | Y | Y |  | Y | Y | Y | Y |  | Y |
|  | 1.2. Are the qualitative data collection methods adequate to address the research question? | Y |  | Y | Y |  |  | Y |  |  | Y | Y |  | Y | Y | Y | Y |  | Y |
|  | 1.3. Are the findings adequately derived from the data? | Y |  | Y | Y |  |  | Y |  |  | Y | Y |  | Y | c/t | Y | Y |  | Y |
|  | 1.4. Is the interpretation of results sufficiently substantiated by data? | Y |  | Y | c/t |  |  | Y |  |  | Y | N |  | Y | N | Y | Y |  | Y |
|  | 1.5 Is there coherence between qualitative data sources, collection, analysis and interpretation? | Y |  | Y | c/t |  |  | Y |  |  | Y | N |  | Y | N | Y | Y |  | Y |
| **4. Quantitative descriptive** | 4.1. Is the sampling strategy relevant to address the research question? |  |  |  |  |  | Y |  |  |  |  |  |  |  |  |  |  | Y |  |
|  | 4.2. Is the sample representative of the target population? |  |  |  |  |  | Y |  |  |  |  |  |  |  |  |  |  | Y |  |
|  | 4.3. Are the measurements appropriate? |  |  |  |  |  | Y |  |  |  |  |  |  |  |  |  |  | Y |  |
|  | 4.4. Is the risk of nonresponse bias low? |  |  |  |  |  | c/t |  |  |  |  |  |  |  |  |  |  | N |  |
|  | 4.5. Is the statistical analysis appropriate to answer the research question? |  |  |  |  |  | Y |  |  |  |  |  |  |  |  |  |  | Y |  |
| **5. Mixed methods** | 5.1. Is there an adequate rationale for using a mixed methods design to address the research question? |  | Y |  |  | Y |  |  | Y | Y |  |  | Y |  |  |  |  |  |  |
|  | 5.2. Are the different components of the study effectively integrated to answer the research question? |  | Y |  |  | Y |  |  | Y | Y |  |  | Y |  |  |  |  |  |  |
|  | 5.3. Are the outputs of the integration of qualitative and quantitative components adequately interpreted? |  | Y |  |  | Y |  |  | Y | Y |  |  | Y |  |  |  |  |  |  |
|  | 5.4. Are divergences and inconsistencies between quantitative and qualitative results adequately addressed? |  | c/t |  |  | Y |  |  | Y | Y |  |  | Y |  |  |  |  |  |  |
|  | 5.5 Do the different components of the study adhere to the quality criteria of each tradition of the methods involved? |  | N |  |  | N |  |  | N | Y |  |  | Y |  |  |  |  |  |  |
|  | **TOTALS** | 5 | 3 | 5 | 2 | 4 | 4 | 5 | 4 | 5 | 5 | 3 | 5 | 5 | 2 | 5 | 5 | 4 | 5 |

Note: c/t=can’t tell

**Table 2: MMAT scores for stakeholders’ views studies - continued**

| **Category of study designs** | **Methodological quality criteria** | Du Mont (2014) | Ericksen (2002) | Fong (2016) | Goddard (2015) | Harvey (2014) | Holton (2018) | Lippert (2008) | Lovett (2004) | Maier (2012) | Mathews (2013) | Musgrave (2014) | Olsen (2017) | Robinson (2009) | Robinson (2011) | Ruch (1980) | Schönbucher (2009) |
| --- | --- | --- | --- | --- | --- | --- | --- | --- | --- | --- | --- | --- | --- | --- | --- | --- | --- |
| **Screening questions (for all types)** | S1. Are there clear research questions? | Y | Y | Y | Y | Y | Y | Y | Y | Y | Y | Y | Y | Y | Y | Y | Y |
|  | S2. Do the collected data allow to address the research questions? | Y | Y | Y | Y | Y | Y | Y | Y | Y | Y | Y | Y | Y | Y | Y | Y |
| **1. Qualitative** | 1.1 Is the qualitative approach appropriate to answer the research question? |  | Y | Y |  |  | Y |  |  | Y |  |  |  |  |  |  |  |
|  | 1.2. Are the qualitative data collection methods adequate to address the research question? |  | Y | Y |  |  | Y |  |  | Y |  |  |  |  |  |  |  |
|  | 1.3. Are the findings adequately derived from the data? |  | Y | Y |  |  | Y |  |  | Y |  |  |  |  |  |  |  |
|  | 1.4. Is the interpretation of results sufficiently substantiated by data? |  | Y | Y |  |  | Y |  |  | Y |  |  |  |  |  |  |  |
|  | 1.5 Is there coherence between qualitative data sources, collection, analysis and interpretation? |  | Y | Y |  |  | Y |  |  |  |  |  |  |  |  |  |  |
| **4. Quantitative descriptive** | 4.1. Is the sampling strategy relevant to address the research question? |  |  |  |  |  |  |  |  |  |  |  | Y |  |  |  |  |
|  | 4.2. Is the sample representative of the target population? |  |  |  |  |  |  |  |  |  |  |  | Y |  |  |  |  |
|  | 4.3. Are the measurements appropriate? |  |  |  |  |  |  |  |  |  |  |  | Y |  |  |  |  |
|  | 4.4. Is the risk of nonresponse bias low? |  |  |  |  |  |  |  |  |  |  |  | Y |  |  |  |  |
|  | 4.5. Is the statistical analysis appropriate to answer the research question? |  |  |  |  |  |  |  |  |  |  |  | Y |  |  |  |  |
| **5. Mixed methods** | 5.1. Is there an adequate rationale for using a mixed methods design to address the research question? | Y |  |  | Y | Y |  | Y | Y |  | Y | Y |  | Y | Y | Y | Y |
|  | 5.2. Are the different components of the study effectively integrated to answer the research question? | Y |  |  | Y | Y |  | Y | Y |  | Y | Y |  | Y | Y | c/t | Y |
|  | 5.3. Are the outputs of the integration of qualitative and quantitative components adequately interpreted? | Y |  |  | Y | Y |  | Y | Y |  | Y | Y |  | Y | Y | c/t | Y |
|  | 5.4. Are divergences and inconsistencies between quantitative and qualitative results adequately addressed? | Y |  |  | Y | Y |  | Y | Y |  | Y | Y |  | Y | Y | c/t | Y |
|  | 5.5 Do the different components of the study adhere to the quality criteria of each tradition of the methods involved? | N |  |  | N | Y |  | N | Y |  | Y | Y |  | Y | c/t | N | Y |
|  | **TOTALS** | 4 | 5 | 5 | 4 | 5 | 5 | 4 | 5 | 5 | 5 | 5 | 5 | 5 | 4 | 1 | 5 |

Note: c/t=can’t tell

**References**

Acierno R, Resnick HS, Flood A, Holmes M. An acute post-rape intervention to prevent substance use and abuse. Addictive Behaviors. 2003;28(9):1701-15. doi: 10.1016/j.addbeh.2003.08.043.

Ahrens C, Campbell R, Wasco S, Aponte G, Grubstein L, Davidson II W. Sexual Assault Nurse Examiner (SANE) programs: Alternative systems for service delivery for sexual assault victims. Journal of Interpersonal Violence. 2000;15(9):921-43. doi: 10.1177/088626000015009002.

Belew S. A program evaluation for the mental health department of the Chicago Children's Advocacy Center [Dissertation]. Illinois, United States: The Chicago School of Professional Psychology; 2012.

Bows H. Practitioner Views on the Impacts, Challenges, and Barriers in Supporting Older Survivors of Sexual Violence. Violence Against Women. 2018;24(9):1070-90. doi: 10.1177/1077801217732348. PubMed PMID: 29332552.

Brooker C, Durmaz E. Mental health, sexual violence and the work of Sexual Assault Referral centres (SARCs) in England. J Forensic Leg Med. 2015;31:47-51. Epub 2015/03/05. doi: 10.1016/j.jflm.2015.01.006. PubMed PMID: 25735784.

Brooker C, Paul S, Sirdifield C. Mental health in Sexual Assault Referral Centres: A survey of forensic physicians. Journal of Forensic & Legal Medicine. 2018;58:117-21. doi: 10.1016/j.jflm.2018.05.008. PubMed PMID: 29800935.

Burton DC, Stanley D, Ireson CL. Child advocacy outreach: using telehealth to expand child sexual abuse services in rural Kentucky. Journal of Telemedicine & Telecare. 2002;8(2):10-2. PubMed PMID: 12217116.

Campbell R, Ahrens CE. Innovative community services for rape victims: an application of multiple case study methodology. American Journal of Community Psychology. 1998;26(4):537-71. doi: <https://doi.org/10.1023/A:1022140921921>. PubMed PMID: 9772731.

Campbell R, Greeson MR, Fehler-Cabral G. With care and compassion: adolescent sexual assault victims' experiences in Sexual Assault Nurse Examiner programs. Journal of Forensic Nursing. 2013;9(2):68-75. doi: 10.1097/JFN.0b013e31828badfa. PubMed PMID: 24158127.

Campbell R, Townsend SM, Long SM, Kinnison KE, Pulley EM, Adames SB, et al. Organizational characteristics of Sexual Assault Nurse Examiner programs: results from the national survey project. Journal of Forensic Nursing. 2005;1(2):57-64, 88. PubMed PMID: 17089484.

Campbell R, Townsend SM, Long SM, Kinnison KE, Pulley EM, Adames SB, et al. Responding to sexual assault victims' medical and emotional needs: a national study of the services provided by SANE programs. Res Nurs Health. 2006;29(5):384-98. Epub 2006/09/16. doi: 10.1002/nur.20137. PubMed PMID: 16977639.

Clark MR, Nackerud L, Larrison CR, Neiderman R. Assessing the congruence between conceptualization and implementation of the collaborative objective in a child sexual abuse response team. Journal of Child Sexual Abuse. 1998;7(2):47-63. doi: 10.1300/J070v07n02_03. PubMed PMID: 107183141.

Cole J, Logan TK. Sexual assault response teams' responses to alcohol-using victims. Journal of Forensic Nursing. 2008;4(4):174-81. doi: 10.1111/j.1939-3938.2008.00029.x.

Cole JE. Factors influencing interprofessional collaboration on sexual assault response teams(SART) [Dissertation]. Lexington, KY: University of Kentucky; 2007.

Comparing Sexual Assault Interventions (COSAI). Mapping the current situation: Findings from the telephone interviews with stakeholders. European Union DAPHNE III Programme 2007-2013, 2012 May.

Cowley R, Walsh E, Horrocks J. The role of the Sexual Assault Nurse Examiner in England: nurse experiences and perspectives. J Forensic Nurs. 2014;10(2):77-83. Epub 2014/05/23. doi: 10.1097/JFN.0000000000000026. PubMed PMID: 24847871.

Downing NR, Mackin ML. The perception of role conflict in sexual assault nursing and its effects on care delivery. J Forensic Nurs. 2012;8(2):53-60. Epub 2012/05/25. doi: 10.1111/j.1939-3938.2012.01135.x. PubMed PMID: 22621663.

Du Mont J, Macdonald S, White M, Turner L, White D, Kaplan S, et al. Client satisfaction with nursing-led sexual assault and domestic violence services in Ontario. J Forensic Nurs. 2014;10(3):122-34. Epub 2014/08/22. doi: 10.1097/JFN.0000000000000035. PubMed PMID: 25144583.

Du Mont J, Parnis D. The doctor's dilemma: caregiving and medicolegal evidence collection. Medicine & Law. 2004;23(3):515-29. PubMed PMID: 15532945.

Du Mont J, White D, McGregor MJ. Investigating the medical forensic examination from the perspectives of sexually assaulted women. Soc Sci Med. 2009;68(4):774-80. Epub 2008/12/20. doi: 10.1016/j.socscimed.2008.11.010. PubMed PMID: 19095341.

Ericksen J, Dudley C, McIntosh G, Ritch L, Shumay S, Simpson M. Clients’ experiences with a specialized sexual assault service. Journal of Emergency Nursing. 2002;28(1):86-90. doi: 10.1067/men.2002.121740.

Fong HF, Bennett CE, Mondestin V, Scribano PV, Mollen C, Wood JN. Caregiver perceptions about mental health services after child sexual abuse. Child Abuse Negl. 2016;51:284-94. Epub 2015/11/26. doi: 10.1016/j.chiabu.2015.09.009. PubMed PMID: 26602155.

Goddard A, Harewood E, Brennan L. Review of pathway following sexual assault for children and young people in London. NHS England, The Havens, Kings College Hospital London; 2015.

Harvey S, Mitchell M, Keeble J, McNaughton Nicholls C, Rahim N. Barriers Faced by Lesbian, Gay, Bisexual and Transgender People in Accessing Domestic Abuse, Stalking and Harassment, and Sexual Violence Services. Welsh Government, 2014.

Holton G, Joyner K, Mash R. Sexual assault survivors' perspectives on clinical follow-up in the Eden District, South Africa: A qualitative study. Afr J Prim Health Care Fam Med. 2018;10(1):e1-e7. Epub 2018/06/27. doi: 10.4102/phcfm.v10i1.1631. PubMed PMID: 29943600; PubMed Central PMCID: PMCPMC6018593.

Lippert T, Favre T, Alexander C, Cross TP. Families who begin versus decline therapy for children who are sexually abused. Child Abuse Negl. 2008;32(9):859-68. Epub 2008/10/28. doi: 10.1016/j.chiabu.2008.02.005. PubMed PMID: 18950858.

Lovett J, Regan L, Kelly L. Sexual Assault Referral Centres : developing good practice and maximising potentials. London: Home Office Research Study 285, 2004.

Maier SL. Sexual assault nurse examiners' perceptions of their relationship with doctors, rape victim advocates, police, and prosecutors. J Interpers Violence. 2012;27(7):1314-40. Epub 2011/12/29. doi: 10.1177/0886260511425242. PubMed PMID: 22203620.

Mathews S, Abrahams N, Jewkes R. Exploring mental health adjustment of children post sexual assault in South Africa. J Child Sex Abus. 2013;22(6):639-57. Epub 2013/08/09. doi: 10.1080/10538712.2013.811137. PubMed PMID: 23924175.

Miller KE, Cranston CC, Davis JL, Newman E, Resnick H. Psychological Outcomes After a Sexual Assault Video Intervention: A Randomized Trial. J Forensic Nurs. 2015;11(3):129-36. Epub 2015/08/21. doi: 10.1097/JFN.0000000000000080. PubMed PMID: 26291847.

Musgrave S, Pickup L, Maskrey V, Blyth A, Notley C, Holland R. A Health Needs Assessment for complainants of Sexual Assault in Norfolk and Suffolk UK: University of East Anglia, Norfolk Constanbulary, Suffold Constanbulary, 2014.

Nixon RDV, Best T, Wilksch SR, Angelakis S, Beatty LJ, Weber N. Cognitive Processing Therapy for the Treatment of Acute Stress Disorder Following Sexual Assault: A Randomised Effectiveness Study. Behaviour Change. 2016;33(4):232-50. doi: 10.1017/bec.2017.2.

Olsen A, Majeed-Ariss R, Teniola S, White C. Improving service responses for people with learning disabilities who have been sexually assaulted: An audit of forensic services. British Journal of Learning Disabilities. 2017;45(4):238-45. doi: 10.1111/bld.12200.

Resnick H, Acierno R, Waldrop AE, King L, King D, Danielson C, et al. Randomised controlled evaluation of an early intervention to prevent post-rape psychopathology. Behav Res Ther. 2007;45(10):2432–47.

Rheingold AA, Danielson CK, Davidson TM, Self-Brown S, Resnick H. Video Intervention for Child and Caregiver Distress Related to the Child Sexual Abuse Medical Examination: A Randomized Controlled Pilot Study. Journal of Child and Family Studies. 2013;22(3):386-97. doi: 10.1007/s10826-012-9591-3.

Robinson A, Hudson K. Different yet complementary: Two approaches to supporting victims of sexual violence in the UK. Criminology & Criminal Justice. 2011;11(5):515-33. doi: 10.1177/1748895811419972.

Robinson AL, Hudson K, Brookman F. A Process Evaluation of Ynys Saff, the Sexual Assault Referral Centre in Cardiff. Wales: University of Cardiff, 2009.

Ruch LO, Chandler S. An Evaluation of a Center for Sexual Assault Victims. Women & Health. 1980;5(1):45-64. doi: 10.1300/J013v05n01_05.

Schonbucher V, Kelly L, Horvath M. Archway: Evaluation of the pilot Scottish rape and sexual assault referral center. UK: London Metropolitan University, Child and Woman Abuse Studies Unit; 2009.

Walsh K, Gilmore AK, Frazier P, Ledray L, Acierno R, Ruggiero KJ, et al. A Randomized Clinical Trial Examining the Effect of Video-Based Prevention of Alcohol and Marijuana Use Among Recent Sexual Assault Victims. Alcoholism: Clinical & Experimental Research. 2017;41(12):2163-72. doi: 10.1111/acer.13505. PubMed PMID: 126530613.
