# Supplementary material for: The identification and treatment of mental health and substance misuse problems in sexual assault services: A systematic review
Source: PLoS One. 2020 Apr 10;15(4):e0231260. doi: 10.1371/journal.pone.0231260 (PMC7147790; doi:10.1371/journal.pone.0231260)
Supplement: S4 File — (DOCX) [file pone.0231260.s004.docx]

**Additional file 4_Q1 Summary tables**

# **Table 1. Identification**

| **Reference** | **Service** | **Service Model** | **Unstructured or unspecified assessment** | **Structured assessment using unspecified or not validated measures** | **Structured assessment using validated standardised measures** | **Verbatim Quotes** |
| --- | --- | --- | --- | --- | --- | --- |
| Beauchamp 2000 | Children first, Child Advocacy Centre (CAC), Louisville, Kentucky, US | CAC | Yes |  |  | All children and families seen were provided crisis intervention services ranging from one session mental health screening to intensive crisis intervention therapy, which could last up to approximately two months. |
| Bechtel 2008 | Paediatric Emergency Department (PED) of Yale-New Haven Children’s Hospital, US | SANE-SART | Yes |  |  | **Contacted Author:** “*Psychosocial evaluation by PED social worker. The social workers conducted behavioural health screening and provided supported counselling in the emergency department (ED). The screening was based on individual social worker’s practice.”* |
| Belew 2012 | Chicago Children's Advocacy Centre (CCAC), US | CAC |  | Yes |  | Trauma/mental issues were identified using a mixture of outcome measures and clinical interviewing. |
| Bennett 2007 | 16 unspecified Child and Youth Protection Programs (CYPPs) based in 16 Paediatric academic health science centres, Canada | Other | Yes |  |  | Nine centres consisted of SANE teams and 5 included on-call physicians. All CYPPs provided psychosocial assessments. **Contacted Author:** *“These were mainly mental health support by social workers.”* |
| Bicanic 2014 | Sexual assault centre, University Medical Centre, Utrecht, Netherlands | SAC (Nordic countries) | Yes |  |  | ‘‘Watchful waiting protocol’’ in the first month following assault." |
| Boykins 2003 | Memphis Sexual assault resource centre, US | SANE-SART | Yes |  |  | Question asked 'do you have a current mental illness' |
| Brooker 2015 | 37 unspecified SARCs, England | SARC (UK) | Yes |  |  | Under half of SARCs routinely assessed mental health and substance misuse issues were not always included. The assessment was completed by a forensic medical examiner (FME). |
| Brown 2013 | Sexual Assault/Domestic Violence Care Centre (SA/DVCC) at Women's College Hospital (WCH) in Ontario, Canada | SA/DVTC (Canada) | Yes |  |  | Medical history taking included documentation of any pre-existing physical or mental illnesses. |
| Campbell 2007 | The Haven, Whitechapel, London, England | SARC (UK) |  | Yes |  | **Standard practice:** All clients having a forensic examination at the Haven were routinely asked whether they have a past or current history of ‘learning difficulties’, ‘deliberate self-harm (DSH)’ or a ‘psychiatric history’. This history was self-identified and in line with current pro-forma used by the Haven and the Association of Forensic Physicians.  **New screening process was developed and implemented:** Questions were asked in relation to psychiatric history (history of anxiety, depression and contact with mental health services, for example), self-harm history (number of incidents and methods used, for example) current suicidal ideation and a mini mental assessment. |
| Chomba 2010 | One-stop centre at the University Teaching Hospital, Zambia | Other |  |  | Yes | After blood tests and post exposure prophylaxis (PEP) administration, the intake forms and the questionnaire for assessment of level of trauma were completed by the nurse or social worker. Mental health assessments for the youth included the post-traumatic stress disorder— reaction index, the strengths and difficulties questionnaire, and my feelings about the abuse. This last measure specifically examined the construct of shame, which is considered to be critical in the Zambian culture. The mental health assessment administered to the caregivers about the abused child is the child behaviour checklist. |
| Creighton 2012 | Lancashire Sexual Assault Forensic Examination (SAFE) centre, England | SARC (UK) | Yes |  |  | Mental health history taking; asking about current prescriptions |
| Darnell 2015 | Harborview Medical Centre, Seattle, Washington, US | SANE-SART | Yes |  |  | ED social workers documented relevant clinical characteristics and SANEs, based on patient self-report, included whether there was evidence that patients had a current mental illness, prior mental health condition, and/or prior trauma history. |
| Du Mont 2004 | 15 Sexual Assault Care and Treatment Centres(SACTCs), Ontario, Canada | SACTC (Australia) |  | Yes |  | General Examination Form (for documenting physical injuries to the victim and her/his emotional state) |
| Durmaz 2014 | Glade SARC, Worcester, England | SARC (UK) | Yes |  |  | Risk assessment for self-harm or any safeguarding concerns. Following the examination, the SARC team will discuss the type of support most appropriate for the victim. |
| Edinburgh 2008 | Unspecified hospital-based Child Advocacy Centre (CAC), US | CAC |  | Yes |  | Victims who were seen in the hospital-based CAC were significantly more likely to have charted evidence of general health assessments recommended for adolescents, as well as screening for psychological symptoms. For example, CAC cases were more likely to have documented evaluations of self-mutilation, history of suicidal ideation and attempts, prior sexual abuse history, prior psychiatric hospitalizations, and existing mental health diagnoses such as Attention Deficit Hyperactivity Disorder (ADHD) or depression, as well as past medical histories of other conditions. Additionally, the CAC cases were significantly more likely to have been assessed for substance use at the time of the assault. |
| Edinburgh 2015 | Unspecified hospital-based CAC, US | CAC |  |  | Yes | Prior to health assessment, youth completed a self-administered questionnaire with items about health behaviours, risk behaviours, and various scales related to supportive family and school and other adult relationships, as well as measures of trauma symptoms and problem substance use. The assessment items include questions from the Minnesota Student Survey (Minnesota Departments of Health and Education), such as a validated measure of problem substance use based on the DSM-IV criteria; the UCLA PTSD trauma screen which was only implemented from 2011 on; and the Child’s Report of Parenting Behaviour Inventory support and control subscales. These assessments were considered part of routine patient care. The psychometric properties of these measures have been evaluated in a number of studies, including with this group. |
| Elliott 1994 | Harbor-UCLA’s Sexual Abuse Crisis Centre, US | Other |  |  | Yes | During 1992 and 1993, 399 children between the ages of 8 and 15 were seen at Harbor-UCLA’s Sexual Abuse Crisis Centre for a forensic evaluation regarding allegations of sexual abuse. Each child was evaluated by a multidisciplinary team that included a licensed mental health clinician, a medical examiner, and either a law enforcement officer or a member of the Department of Children Services (or both). The evaluation included: (a) at least one interview with the minor, (b) a medical examination, (c) whenever possible, an interview of the primary non-offending caretaker, and (d) an assessment of the child’s psychological distress using the Trauma Symptom Checklist for Children (TSCC). |
| Guney 2018 | Izmir CAC, Turkey | CAC |  | Yes |  | Psychiatric evaluation appointment with Psychiatrist. Psychiatric examinations of CSA victims were evaluated by using Diagnostic and Statistical Manual of Mental Disorders-V criteria. Mental health examinations initiated in the following 10 days after the statement was taken at the CAC. |
| Hagemann 2013 | Sexual Assault Centre, St. Olavs University Hospital, Trondheim, Norway | SAC (Nordic countries) |  |  | Yes | Psychosocial history was registered at the service. The hospital’s record data includes all information collected at the consultation at the sexual assault centre in the acute phase (self-reported and reported by a possible companion or from the referral note / phone referral if present), in addition to available follow-up data. Follow-up was performed by a psychiatric SAC nurse, and she asked the patients regarding prior mental health problems, drug abuse and prior psychosocial care/treatment.  **Contacted Author:***“The follow-up service (by a psychiatric nurse at the SAC) was for all patients who consents to it, but those who already have a well-established psychological support contact, were only seen (or talked to them on the phone) for a short period of time (for possible injury control, information of the test-results or for re-testing for STI, pregnancy, vaccine (hepatitis B) etc.). The other patients may come for up to five conversations.PTSS-12 was used for the follow-up assessment. This tool scores dichotomously: sleep problems, night mares about the event, a feeling of a depressed condition, nervous or jumpy by a sudden noise or movement, a tendency to isolate, irritation, feelings fluctuating up and down, bad conscious/self-blame, fear of situations reminding of the event, feeling of a tensed body, memory problems, difficulties to concentrate*.” |
| Heke 2009 | The Havens, London, England | SARC (UK) |  | Yes |  | The paper was about an audit of risk identification tools and management pathways developed for the Havens. The identification tool comprised of a risk identification form completed at first contact with the service and at follow-up. Risk management included referral to A&E, Internal referral for clinical psychology or external referral to other services. |
| Lurie 2013 | First Regional Israeli Centre for Sexual Assault Victims, Israel | Other |  | Yes |  | The gynaecologist and social worker document data in a data collection form including data on: Mental health status, psychiatric background and history of involvement with social care services. |
| Manning 2019 | St. Mary’s SARC, Manchester, England | SARC (UK) |  | Yes |  | During the forensic medical examination (FME) a full medical history is taken by the forensic physician, including a psychiatric history, followed by a mental state examination which includes an assessment of suicide and deliberate self-harm risk. Counselling is also available at the Centre |
| Miller 2015 | SANE program at a hospital, South Carolina, US | SANE (US) | Yes |  |  | **Contacted Author:** *“There is not routine mental health screening. The SANE nurse asks about past health history. There is where clients tell them of their mental health diagnoses and what meds they take or are supposed to take. The SANE, also, asks if they have been previously victimized and that brings in more history. They, also, ask about suicidal ideation.”* |
| Musgrave 2014 | The Ferns centre in Suffolk and the Harbour Centre in Norfolk, England | SARC (UK) |  | Yes |  | Vulnerabilities including mental health and drug and alcohol use were recorded in the clinical form completed during the FME. |
| Nelson 2016 | The Sexual Abuse  Treatment Centre (SATC), Kapi' Olani, Hawaii | Other |  | Yes |  | Clients are assessed using the Teen and Adult Counselling Evaluation form which gives a score for 'coping' and level of 'PTSD'. |
| Robinson 2009 | Ynys SAFF, Cardiff, Wales | SARC (UK) | Yes |  |  | General question asked about mental health history during examination |
| Sacks 2008 | The Haven Whitechapel, London, England | SARC (UK) |  | Yes |  | SARC clients were screened using a Risk Assessment Proforma to identify mental health issues. |
| Speck 2005 | 1. Government SANE program, Freehold, New Jersey 2. Commercial SANE program, Los Angeles/ Orange County, California 3. Non-profit SANE program, Athens, Georgia | SANE-SART | Yes |  |  | (1&2) The patient evaluation included mental health history and voluntary drug use  (3)The patient evaluation included mental health |
| Vik 2016 | St. Olavs University Hospital , Trondheim, Norway | SAC (Nordic countries) | Yes |  |  | Asking a question about current mental health and using drug prescriptions as a proxy. |
| Winters 2011 | SAFE Place Merseyside (SPM), Liverpool, England | SARC (UK) | Yes |  |  | SPM clients were routinely asked if they have a disability. Out of 531 clients giving an answer to this question 20.5% said they had a disability and 422 (79.5%) declared they had none. Of those answering that they had a disability, the highest proportion 48 (44%) had a mental health condition, although this only represented 9% of the whole population. |
| Zijlstra 2017 | Sexual and family violence, Nijmegen (CSFV), Netherlands | Other |  |  | Yes | A case manager, based at the academic Primary Health Care Centre, calls victims the day after acute care has been provided. This case manager is in charge of psychosocial follow-up care such as providing psychoeducation and referral to psychosocial or legal help, if necessary. Follow-up care can be done by phone or in face-to-face contacts, depending on the victims' preferences.  **Contacted Author:** *“The case manager screens for PTSD at one month and three months after the incident according to the NICE guidelines. The Trauma screening questionnaire (TSQ) is used for people over 18 years old, and the children’s impact of events scale (CRIES) for those under 18 years. The case manager keeps files, registering the victims' needs and action plans. If victims do not want or need follow-up care, the case manager asks for the reason why and registers this as well. After three months, the case manager usually transfers the care to the victims' GP.”* |
| Zilkens 2018 | Sexual Assault Resource Centre (SARC), Perth, Australia | SARC/SAS (Australia) |  | Yes |  | Men underwent a standardised medical examination and data collection by forensically trained doctors  Following consent for general body and/or ano-genital examination. Current mental illness was based on the patient's self-reported history and included psychotic (e.g. schizophrenia, bipolar disorder) and nonpsychotic (e.g. anxiety, depression) disorders.  **Contacted Author:** *“The service has its standard proforma or notes and as of the routine or regular history, every patient is asked if they have any mental health history or concerns. The patient may say that they have depression, anxiety or schizophrenia, alcoholism, anorexia etc. From that the examining doctor may ask further questions, such as has this been formally diagnosed, that is has the patient been told by a doctor or psychiatrist that there is a diagnosis, are they taking medication or seeing a professional etc.”* |

# **Table 2. Support within SARCs**

| **Reference** | **Service** | **Service Model** | **Immediate emotional support during first SARC contact(s) only** | **Follow-up care provided: supportive and/or unspecified counselling** | **Follow-up care provided: Structured psychological Interventions** | **Mental health support provided(type unclear)** | **No mental health support provided (provisional category)** | **Verbatim Quotes** |
| --- | --- | --- | --- | --- | --- | --- | --- | --- |
| Abrahams 2010 | Sinawe sexual assault centre, Mthatha, Eastern Cape, South Africa | Other |  |  |  | Yes |  | Psychological support for patients and court preparation was available from the social worker or, if necessary, the hospital psychiatric service. |
|  | Karl Bremer sexual assault service, Cape Town, Western Cape, South Africa | Other |  |  |  |  | Yes | The service at the centre was exclusively medical and rape survivors are referred to volunteer counsellors. There was no NGO working on rape in the area. |
| Ahrens 2000 | Young Women’s Christian Association(YWCA) SANE, Michigan, US | SANE |  | Yes |  |  |  | When a victim arrives support is offered by a sexual assault victim advocate. On completion of the FME the trained volunteer advocate informs the victim of the available counselling services and obtains permission to have the volunteer coordinator contact them by phone within 2 days to offer an appointment for counselling. Second follow-up contact 2-3 weeks after the examination to see if the client has followed through with treatment and counselling. |
|  | Battle Creek Health System (BCHS) SANE, Michigan, US | SANE |  | Yes |  |  |  | On completion of the FME, the SANE and the advocate provide verbal and written info on the emotional consequences of the assault and emphasize the importance of follow-up care. Advocates and counselling staff contact victims by phone within 48 hrs and re-emphasize the importance of follow-up counselling. |
| Beauchamp 2000 | Children first, CAC, Louisville, Kentucky, US | CAC |  | Yes |  |  |  | All children and families seen are provided crisis intervention services ranging from one session mental health screening to intensive crisis intervention therapy which can last up to approximately two months. At the end of treatment at Children First, the child/family will either be terminated from treatment altogether or referred to other community agencies or private therapists who can provide longer term interventions if necessary. The Children First professional staff consists of mental health therapists, a clinical supervisor, a child and adolescent gynaecological physician, a registered nurse, and a medical assistant. |
| Bechtel 2008 | Paediatric emergency department (PED) of Yale-New Haven Children’s Hospital, US | SANE |  | Yes |  |  |  | Support workers provided supportive counselling in the ED. |
| Belew 2012 | Chicago Children's  Advocacy Centre (CCAC), US | CAC |  |  | Yes |  |  | There were five mental health programs at CCAC that provided direct services to children and their families: the Art Therapy Program, the Adolescent Program, the Crisis Program, the General Mental Health Program, and the Services, Options, and Treatment Program (STOP Program). |
| Bennett 2007 | 16 Child and youth protection programs (CYPPs) based in 16 Paediatric academic health science centres, Canada | CYPPs |  | Yes |  |  |  | 6 out of 12 programs had a psychologist. Most programs provided mental health crisis intervention and brief supportive counselling, but only 3 programs had program staff dedicated to ongoing mental health treatment. |
| Bicanic 2014 | Sexual assault centre, University Medical Centre Utrecht, Netherlands | Not reported |  |  | Yes |  |  | On site treatment by a separate provider with shared health records: “39% of both young and adult patients received either CBT or EMDR therapy, though not immediately; If necessary, evidence-based treatment for PTSD is provided such as cognitive behaviour therapy (CBT) or eye movement desensitisation reprocessing (EMDR) therapy. If the victim is a child, parents or caregivers are offered parallel psychological support, but children at the age of 16 years or older can consent to services at the centre without their parents being notified." |
| Bramsen 2009 | Centre for Rape Victims (CRV), Aarhus, Denmark |  |  | Yes |  |  |  | Rape and sexual assault victims who contact the CRV are automatically referred to a licensed psychologist affiliated with the CRV unless other arrangements have been made. The psychologist offers counseling either individually, in the company of relatives, or in groups. In addition, the psychologist offers to contact and collaborate with social services, the police department, the school, the victim’s family, or any other relevant party whose involvement will be beneficial to the victim. The service is free of charge and unlimited, depending on the needs of each individual case. With the victim’s consent, part of this outreach also consists of a follow-up of people surrounding the victim who may have been emotionally affected or secondarily traumatized by the incident and from whom the victim may need support immediately after the incident or at a later date. Furthermore, a standardized follow-up routine has been established for all victims Model for Treating Victims of Rape and Sexual Assault 895 whereby the psychologist sends a follow-up letter to each victim six months after the end of their treatment. The letter includes a questionnaire, but is also part of the therapeutic care function post discharge. If the victim does not respond to this letter, the psychologist will follow up with a personal phone call. |
| Boykins 2003 | Memphis Sexual assault resource centre, US | SANE |  | Yes |  |  |  | Crisis intervention and individual and group counselling. The service has two full-time counsellors. |
| Brown 2013 | Sexual Assault/Domestic Violence  Care Centre (SA/DVCC) at Women's College Hospital (WCH) in Ontario, Canada | SA/DVCC |  | Yes |  |  |  | At the completion of the emergency visit, the SANE informs the client of on-site follow-up services and arranges an appointment and/or a follow-up phone call. On-site follow-up services include referral to individual and group counselling. On-site counselling services are available on a short-term basis for up to 20 sessions. |
| Campbell 2007 | The Haven, Whitechapel, London, England | SARC (UK) | Yes |  |  |  |  | There were 2 possible routes decided by the examiner to further treatment/support: 1) urgent follow-up (to be seen within 3 days at the Haven) and 2) immediate referral to the psychiatric liaison team in the Accident & Emergency department. Whilst some clients were identified as needing these services, it is not clear what the take-up rates were. |
| Ciancone 2000 | 60 SANE programs, US | SANE |  | Yes |  |  |  | 93% of the programs offer the patient someone other than the examiner to provide emotional support-psychological counselling. In 90% (45/50) of programs, 10% or less of initial examinations require additional examination by a physician. Severe psychological trauma is among the reasons requiring additional examinations. Only one program reported not offering follow-up for psychological counselling |
| Cooper 1982 | Sexual Assault Referral Centre, Perth, Australia | SARC |  | Yes |  |  |  | Victims arriving at the SARC meet the support/social worker that supports them emotionally by providing introductions and explanations of the role of the centre.  Referrals relating to a past sexual assault are seen initially by the support/social worker that provides emotional and practical support, or if necessary referral to another person such as psychiatrist or psychologist for long term counselling. |
| Deller 1979 | SARC, Perth, Australia | SARC |  | Yes |  |  |  | Undescribed counselling. It can be long-term and time-consuming involving the victim and its friends/family. |
| Dhairyawan 2017 | 12 unspecified SARCs, UK | SARC (UK) |  | Yes |  |  |  | For follow-up care, 12/21 (57%) provided in house counselling. |
| Du Mont 2004 | 15 Sexual Assault Care and Treatment Centres(SACTCs), Ontario, Canada | SACTC (Australia) | Yes |  |  |  |  | The on-call sexual assault physician provides a number of services, in conjunction with the on-call nurse. They crisis counsel and offer emotional support to the victim, explain available treatment and forensic options to her/him, and provide appropriate referrals to support services operating within the community. They also offer comprehensive medical care including a complete physical examination. |
| Du Mont 2014 | 30 unspecified SA/DVTCs, Ontario, Canada | SA/DVTCs |  | Yes |  |  |  | On-site follow-up care and crisis counselling, and referral to community agencies for ongoing support. |
| Duddle 1991 | St Mary’s SARC, Manchester, England | SARC (UK) |  | Yes |  |  |  | At the centre there are trained counsellors on call 24 hours a day and they will remain with the woman during the police interrogation, which can take place in the centre. The counsellor offers the client continued counselling for as long as she needs it to overcome problems which can occur later related to the assault. If requested, she will also attend court with the client. This is often a very traumatic part of the whole proceedings bringing back all the original emotions and is often, regrettably, delayed by many months. |
| Durmaz 2014 | Glade SARC, Worcester, England | SARC (UK) |  | Yes |  |  |  | Risk assessment for self-harm or any safeguarding concerns. Following the examination, the SARC team will discuss the type of support most appropriate for the victim. This may involve counselling or more practical support from an independent sexual violence adviser, or follow up with sexual health services or mental health services. |
| Edinburgh 2008 | Unspecified Child Advocacy Centre, US | CAC |  | Yes |  |  |  | In-house psychologist |
| Foley 1990 | St Mary’s SARC, Manchester, England | SARC (UK) |  | Yes |  |  |  | Those with trauma referred to counsellors. Counselling provided in-house (this is 1990 and very early days of the first SARC in the country)  "In line with this, the counsellors at the SARC have been trained in crisis Intervention and psycho-sexual counselling techniques. Both forms of counselling are based on a specific understanding of 'normality' and specific understandings of 'recovery'" and more mentions of counselling.” |
| Gibney 2014 | Lancashire Sexual Assault Forensic Examination (SAFE) centre, England | SARC (UK) |  |  |  | Yes |  | Complainants are seen and examined at the centre, and appropriate follow up arranged; be it with Genito-Urinary Medicine, Paediatrician referral, Social Services or via General Practitioner correspondence. Two dedicated Independent Sexual Violence Advocates (ISVA) follow up those seen at the centre, should they wish to access their support. One of these ISVAs is specifically for adults; the other is a Children's and Young Person's Advocate (CYPA), seeing children and their parents/carers, offering emotional and practical support as well as advising parents/carers of the process after attending the centre and of the criminal proceedings. They do not offer counselling but signpost or refer on to other services if psychological therapy or counselling is requested. The follow up for psychological support however, is less clear cut and the case below highlights how difficult organising psychological support can be. |
| Goddard 2015 | The Havens, London, England | SARC (UK) |  |  |  |  | Yes | The Havens aren’t commissioned to provide counselling for children under 18 years of age |
| Goodyear 1989 | The HELP Centre, Sexual Assault Centre, Auckland, New Zealand | SAC |  |  | Yes |  |  | Counselling is offered on-site. Counselling includes telephone counselling, one-to-one counselling, group work (e.g children's group, adolescent group, young women's group), play therapy and some family therapy. Counsellors are trained and operate in a 24-hour roster basis. |
| Hagemann 2013 | Sexual Assault Centre, St. Olavs University Hospital, Trondheim, Norway | SAC (Nordic countries) |  | Yes |  |  |  | Follow-up was performed by a psychiatric SAC nurse, and she asked the patients regarding prior mental health problems, drug abuse and prior psychosocial care/treatment.  **Contacted Author:***“The follow-up service (by a psychiatric nurse at the SAC) was for all patients who consents to it, but those who already have a well-established psychological support contact, were only seen (or talked to them on the phone) for a short period of time (for possible injury control, information of the test-results or for re-testing for STI, pregnancy, vaccine (hepatitis B) etc.). The other patients may come for up to five conversations.PTSS-12 was used for the follow-up assessment. This tool scores dichotomously: sleep problems, night mares about the event, a feeling of a depressed condition, nervous or jumpy by a sudden noise or movement, a tendency to isolate, irritation, feelings fluctuating up and down, bad conscious/self-blame, fear of situations reminding of the event, feeling of a tensed body, memory problems, difficulties to concentrate*.” |
| Hanson 1985 | Child and Adolescent  Sexual Abuse Resource  Centre (CASARC), San Francisco, US | CASARC |  | Yes |  |  |  | After the initial examination, a CASARC counsellor (a social worker or psychiatric nurse) will be assigned to provide psychological counselling for the child and family for up to two or three months, or until the court proceedings are completed. The centre provides individual sessions as well as therapy groups and family counselling. Part of the CASARC counsellor’s role is to prepare the child for court and to be available as a support during the court proceedings. |
| Henry 1980 | Sexual assault referral centre, Sir Charles Gairdner Hospital, Pert, Australia | SARC/SAS (Australia) |  | Yes |  |  |  | The centre provided counselling with the aim to assist victims and families with any problems occurring as a result of the assault. Assistance may be practical, social, emotional or psychological. The Centre aims not to take on a person's life problems, just those problems related to the sexual assault. The counselling staff consists of one full time social worker who trains and co-ordinates the counselling team and counsels all victims referred during the day. This social worker has back up from the Social Work Department. The other counsellors, of which there are four, work on a sessional basis and are on call from their own homes; they cover out of hours rosters. These counsellors do not have professional qualifications; they are chosen from their warmth and caring approach to people, and are given in-service training on crisis intervention and counselling of rape victims. During initial contact the counsellor spends some time alone with the victim, in order to concentrate on the person's immediate emotional and practical concerns. |
| Heke 2009 | The Havens, London, England | SARC (UK) | . | Yes |  |  |  | The paper was about an audit of risk identification tools and management pathways developed for the Havens. The identification tool comprised of a risk identification form completed at first contact with the service and at follow-up. Risk management included referral to A&E, internal referral for clinical psychology or external referral to other services. |
| Hester 2018 | Unspecified SARC, England | SARC (UK) |  |  |  | Yes |  | The ISVAs provide 'emotional support' |
| Holton 2018 | George Regional Hospital Thuthuzela Care Centre (TCC), Eden District, South Africa | TCC |  |  |  | Yes |  | During office hours, psychosocial support was provided by Thuthuzela staff. After hours, counselling was covered by community partners. Health care services were provided by the hospital’s emergency centre doctors within the Thuthuzela examination room. All follow-up consultations were provided by the hospital’s occupational health and safety professional nurse. |
| Hubel 2014 | Unspecified Midwest CAC, US | CAC |  |  | Yes |  |  | A trained master's level therapist and pre-master's level co-therapists deliver psychological group treatment (Project SAFE). All participating therapists work toward their doctoral degree in clinical psychology. Co-therapists receive training by licensed psychologists and experienced master’s level therapists prior to delivery of Project SAFE services.  Project SAFE (Sexual Abuse Family Education), is a 12 session, parallel (children and non-offending caregiver groups meet separately but concurrently), manualized, cognitive behavioural group treatment for children who have experienced CSA and their non-offending caregivers. The program was developed at the University of Nebraska-Lincoln and began delivering on-site mental health services at the CAC in 2000. Treatment is provided to groups of children with varied levels and types of symptoms simultaneously. It is designed to improve outcomes for children’s sense of stigmatization and isolation associated with the abuse, to assist them in exploring and coping with their feelings about the abuse, and to empower them in preventing future victimization. It is delivered weekly and each session lasts 90 minutes. |
| Kerr 2003 | The Haven, London, England | SARC (UK) |  | Yes |  |  |  | When someone presents for examination following sexual assault, a supportive environment is essential, but formal counselling is inappropriate. Crisis workers provide immediate psychological support. At follow-up the needs of clients are assessed and short term counselling is offered by health advisers. The follow-up clinic offers psychosocial support and short-term counselling at the Haven. Clients with needs that cannot be provided within the service are referred on, either locally or in their district of residence. Some clients will only wish to access counselling months or years later, and so written information about potential later sources of help is also provided. Follow-up visit is scheduled 6-10 days after the assault to screen for STIs, provide medical care and psychosocial support. The visit can take place at the Haven, or elsewhere if more convenient for the client. Crisis worker provide support to the complainant, but also assist the doctor with the examination. The doctor and crisis worker involved in the forensic examination are not involved in the follow up. |
| Kille 1986 | Sexual assault referral service at the North-Western General Hospital, Burnie, Tasmania | SARS |  | Yes |  |  |  | Hospital colleagues including psychologists and psychiatrists are available for consultation. Victims may be seen by social workers or counsellors. Counselling is provided. |
| Larsen 2016 | Centre for Victims of Sexual Assault, Copenhagen, Denmark | SAC (Nordic countries) |  | Yes |  |  |  | The centre has a psychologist and offers social counselling, and psychotherapy. |
| Ledray 1993 | Sexual Assault Resource Service (SARS), Minneapolis, Minnesota, US | SARS |  | Yes |  |  |  | Crisis intervention and long-term counselling |
| Lippert 2008 | The Dallas Children’s Advocacy Centre, US | DCAC |  | Yes |  |  |  | A family’s first visit to DCAC is generally for a forensic interview of a child (i.e., an investigative interview to assess the truth of allegations about abuse). After the interview, the child is referred for therapy if abuse was disclosed or is highly suspected. Children may also be referred to therapy when abuse is suspected and a child is uncooperative with an interview. Family members are referred, too, if they experience significant distress. Per DCAC policy, investigators and the interviewer meet after the interview to plan next actions, with no formal policy specifying who should make the therapy referral and how soon it should be made. When a referral is planned, caregivers are informed that they will receive the referral and instructions through the mail. Caregivers are then mailed a letter citing the referral and its source and describing the process and purpose of therapy, with a request for a telephone reply within 2 weeks. Therapists are assigned for families who respond to the letter. |
| Lovett 2004 | (1)St Mary’s SARC, Manchester, England | SARC (UK) |  | Yes |  |  |  | Immediate crisis support is provided by the crisis worker. At the end of the examination a debriefing session takes place with the crisis worker, where additional St Mary's services are outlined. Since the appointment of the Support Worker all attendees are re - contacted the next working day; she offers both to attend the statement-taking (if it has not already been done) and to facilitate access both to St Mary ’s services and other external organisations. Proactive follow-up is also part of the support worker’s role. Counselling is available on site, and there is no limit set on the number of sessions. St Mary’s also operates an open advice, information and referral service through the telephone, including an ‘out-of-hours’ service. |
|  | (2)REACH, Ellis Fraser Centre, Sunderland, England | SARC (UK) |  | Yes |  |  |  | Procedures are similar to those for St Mary’s, with the exception that the role of the crisis worker has to be fulfilled by a combination of a specialist police officer and the forensic doctor. Counselling services are available on site and are limited to a set number of ten sessions, although there is some flexibility in individual cases. REACH also offers group counselling in some cases, and is the only SARC to do so currently. |
|  | (3)REACH, Rhona Cross Centre, Newcastle, England | SARC (UK) |  | Yes |  |  |  | Same as REACH 2a |
|  | (4)STAR, West Yorkshire, England | SARC (UK) |  | Yes |  |  |  | Its primary role, therefore has been to co-ordinate and commission local support and counselling for adults, and also offer immediate support and advice through a helpline. Counselling is provided throughout West Yorkshire, on a commissioned basis, by STAR. It is commissioned from accredited counsellors, who are responsible for making arrangements with service users about the location and timing of sessions. The rationale here is that this enables access close to where service users live. A maximum of ten sessions will be paid for by STAR for any service user. Two innovative elements of the STAR model are Initial Support Workers and the Case Tracker. Initial Support Workers are volunteers, trained by STAR, who offer immediate crisis support for six weeks – this may be practical, access to information, as well as just someone to talk to. The preferred model is for service users to move from this more informal support into counselling. The rationale here is that this enables access close to where service users live. A maximum of ten sessions will be paid for by STAR for any service user. Two innovative elements of the STAR model are Initial Support Workers and the Case Tracker. Initial Support Workers are volunteers, trained by STAR, who offer immediate crisis support for six weeks – this may be practical, access to information, as well as just someone to talk to. |
| Manning 2019 | St. Mary’s SARC, Manchester, England | SARC (UK) |  | Yes |  |  |  | During the FME a full medical history is taken by the forensic physician, including a psychiatric history, followed by a mental state examination which includes an assessment of suicide and deliberate self-harm risk. Counselling is also available at the Centre. Both the ISVA and counselling aspects of the service are delivered via a hub and spoke model. |
| McLean 2005 | St Mary’s SARC, Manchester, UK | SARC (UK) |  | Yes |  |  |  | Counselling on-site |
| Minden 1989 | Victim Care Service at the Boston City Hospital, US |  |  | Yes |  |  |  | The service offers up to 4 supportive counselling sessions provided by a team of Masters-prepared psychiatric nurses and social workers. These clinicians act as advocates for the most vulnerable patients and make sure they are hooked up with the necessary other supports and services. Ongoing therapy operates on a referral basis as BCH doesn’t have the capacity to provide outpatient mental health services. The VCS maintains a linkage system with other community programs who offer individual, family and group therapy in order to provide continuity of care for its patients. |
| Nelson 2016 | The Sexual Abuse  Treatment Centre (SATC), Kapi' Olani, Hawaii | SATC |  | Yes |  |  |  | It in-person provides crisis intervention as well as psychotherapy. |
| Nixon 2016 | Yarrow Place Sexual Assault Crisis Centre, Adelaide, Australia | Not reported |  | Yes |  |  |  | Supportive counselling organised within SARC |
| Olsen 2017 | St Mary’s SARC, Manchester, England | SARC (UK) |  | Yes |  |  |  | Counselling is available to all patients who have attended St Mary’s SARC following a sexual assault. Only one person from the learning disability cohort accessed the counselling offered by the centre as compared to 12 patients from the control group. Some patients reported seeking counselling outside the service (two patients from the learning disability group and one from the control group). These figures suggest that people with learning disabilities who have experienced sexual assault or rape were 50% less likely to access counselling than people without learning disabilities. |
| Pillai 2006 | 10 unspecified SARCs, UK | SARC (UK) |  | Yes |  |  |  | Follow up counselling support was available in 10 of 12 SARCs (83%). Sixteen of 49 non SARC services (33%) who answered this indicated they could offer follow up counselling, but in many it was not clear whether this was by referral to an outside agency. West Yorkshire is exceptional in having the well-established STAR service. |
| Ranney 2010 | Centre for assault recovery-Eldoret (CARE), Kenya, Africa | CARE |  | Yes |  |  |  | Only 44% of patients were documented as receiving counselling. This low rate is most likely due to the fact that the entire A&E department had one counsellor, who was present only 40 h per week. |
| Rheingold 2013 | Child Advocacy Centre, South East region of the US | CAC | Yes |  |  |  |  | Rape crisis advocate offered to support child and family through the medical examination process. Signposting by rape crisis advocate |
| Robinson 2009 | Ynys SAFF, Cardiff, Wales, UK | SARC |  |  |  | Yes |  | Emotional support (crisis intervention / ongoing non-therapeutic / advocacy) is provided by the crisis workers or ISVAs. The ISVAs offer crisis intervention, ongoing non-therapeutic emotional support, and practical assistance and advice to all clients accessing the SARC. The emotional support they provide is distinctly different to counselling and consists of exploring coping strategies, assessing safety, well-being and providing information and help with responding to trauma and anxiety. However, the ISVAs will, if required, signpost clients on to other counselling and support services. |
| Ruch 1980 | Sexual assault treatment centre, Western US | SATC |  | Yes |  |  |  | All victims are accompanied through the treatment process at the hospital by the SATC staff social worker or crisis worker, who provides them with counselling, information, advocacy, and emotional support. |
| Sacks 2008 | The Haven Whitechapel, London, England | SARC (UK) |  | Yes |  |  |  | A retrospective case-note review of 58 female patients aged between 13 and 18. The service provides in-house mental health support(counselling or clinical psychology), but also refers to adolescent mental health services with active follow-up |
| Schei 2003 | Centre for Victims of Sexual Assault, Copenhagen, Denmark | SAC (Nordic countries) |  | Yes |  |  |  | The centre offers a 24-h service, which includes forensic evidence collection and documentation as well as a comprehensive medical treatment and psychological follow up. A specialized team of nurses, gynaecologists, social workers and a psychologist was created. |
| Schonbucher 2009 | Archway Glasgow SARC, Scotland | SARC (UK) |  | Yes |  |  |  | Follow-up services consist of support work and counselling. The support work comprises of advocacy and practical support alongside providing emotional support and accompaniment. The support worker refers people to other follow up services, including counselling. Counselling is envisaged as providing professional support to help the service users cope with the emotional and social consequences, with the aim off minimising negative repercussions on the service users’ mental health. Both counselling and support worker sessions take place in the SARC. |
| Sidenius 2004 | Centre for Victims of Sexual Assault, Copenhagen, Denmark | SAC (Nordic countries) |  |  | Yes |  |  | They have a multi-disciplinary team including social counsellors and psychologists. The Centre offers psychological emergency treatment and short- or long-term psychotherapeutic support. The majority of those who contact the centre accept the offer of psychological help, which usually consists of individual therapy. Group therapy is also offered as well as support and guidance to relatives, who are often deeply affected by these offences. It also offers service users a follow-up appointment a few days later where psychological and social support initiatives are planned. |
| Signal 2013 | Phoenix House, SARC, Queensland, Australia | SARC |  |  | Yes |  |  | In-clinic counselling and Adjunct Equine Facilitated Program (EFT) |
| Speck 2005 | 1. Government SANE program, Freehold, New Jersey 2. Non-profit SANE program, Athens, Georgia 3. Commercial SANE program, Los Angeles/ Orange County, California | SANE |  | Yes |  |  |  | All three services provide an immediate medical triage with referral for medical or psychological stabilization, crisis intervention…,as well as in-house counselling and crisis counselling |
| Ward 2006 | “Rainbo Centres” Freetown, Kailahun, Kenema and Kono districts in Sierra Leone | Not reported |  | Yes |  |  |  | Psychosocial counselling, referral and advocacy services |
| Winters 2011 | SAFE Place Merseyside (SPM), Liverpool, England | SARC (UK) |  |  |  | Yes |  | On arrival at the Centre, clients will be able to access emotional and practical support from a Crisis Worker and will also be given information about how to access further care/counselling and support/advice on various criminal justice options. |
| Zijlstra 2017 | Centre for Sexual and Family Violence (CSFV), Nijmegen, Netherlands | Not reported |  |  |  | Yes |  | A case manager, based at the academic Primary Health Care Centre, calls victims the day after acute care has been provided. This case manager is in charge of psychosocial follow-up care such as providing psychoeducation and referral to psychosocial or legal help, if necessary. Follow-up care can be done by phone or in face-to-face contacts, depending on the victims' preferences. Preferably, the screening is done face to face. Victims are invited to come to our follow up care centre (located in a primary health care centre). If this is not possible, screening is done by phone.  **Contacted Author:** *“The case manager screens for PTSD at one month and three months after the incident according to the NICE guidelines. The case manager keeps files, registering the victims' needs and action plans. If victims do not want or need follow-up care, the case manager asks for the reason why and registers this as well. After three months, the case manager usually transfers the care to the victims' GP.”* |

# **Table 3. Referral-on**

| **Reference** | **Service** | **Service Model** | **Signposting and referral** | **Referral on with active follow-up** | **Verbatim Quotes** |
| --- | --- | --- | --- | --- | --- |
| Abrahams 2010 | Karl Bremer sexual assault service, Cape Town, Western Cape, South Africa | Other | Yes |  | The service at the centre is exclusively medical and rape survivors are referred to volunteer counsellors. There is no NGO working on rape in the area. |
| Beauchamp 2000 | Children first, CAC, Louisville, Kentucky, US | CAC | Yes |  | All children and families seen are provided crisis intervention services ranging from one session mental health screening to intensive crisis intervention therapy which can last up to approximately two months. At the end of treatment at Children First, the child/family will either be terminated from treatment altogether or referred to other community agencies or private therapists who can provide longer term interventions if necessary. The Children First professional staff consists of mental health therapists, a clinical supervisor, a child and adolescent gynaecological physician, a registered nurse, and a medical assistant. |
| Bechtel 2008 | PED of Yale-New Haven Children’s Hospital, US | SANE-SART | Yes |  | Referral to Rape Crisis Centre for short-term mental health support and counselling |
| Belew 2013 | Chicago Children's  Advocacy Centre (CCAC), US | CAC | Yes |  | Another component of the mental health department is the Network of Treatment Providers - Child Sexual Abuse (NTP-CSA) is also facilitated by the CCAC. This NTP-CSA allows the CCAC to connect clients to long-term therapy services post-trauma. This referral base consists of both community agencies and private practitioners who agree to provide post-trauma services to abused children, their siblings, and non-offending caregivers in a manner that is consistent with the CAC model of treatment. One reason for utilizing outside treatment providers is that the CCAC is understaffed in regard to mental health professionals  Once risk and well-being is assessed, the client may be referred for a wide range of treatment options, from outpatient psychotherapy to inpatient hospitalization, depending on the level of psychiatric stabilization exhibited during the screening process. Any mental health professional at CCAC may perform this screening; however, the Mental Health Manager primarily facilitates the Crisis Program. |
| Bennett 2007 | 16 Child and youth protection programs (CYPPs) based in 16 Paediatric academic health science centres, Canada | Other | Yes |  | Eleven programs referred all their cases to community based practitioners for mental health treatment, while three programs referred cases to their own hospital’s mental health services. |
| Boykins 2003 | Memphis Sexual assault resource centre, US | SANE-SART | Yes |  | Referral on for counselling |
| Campbell 2006 | 110 unspecified SANE programs, US | SANE-SART | Yes |  | Most programs consistently provided referrals for mental health and social services (396). 77% services provide referrals for every patient, including to mental health services (388). Table 1 mentions referrals not undertaken by SANE because 'provided by advocate (n=14)' (388) |
| Campbell 2007 | The Haven, Whitechapel, London, England | SARC (UK) | Yes |  | There were 2 possible routes decided by the examiner to further treatment/support: 1) urgent follow-up (to be seen within 3 days at the Haven) and 2) immediate referral to the psychiatric liaison team in the Accident & Emergency department. Whilst some clients were identified as needing these services, it is not clear what the take-up rates were. |
| Campbell 2008 | Unknown Midwestern SANE program, US | SANE | Yes |  | This SANE program also hoped to provide information and linkages to their parent agency’s counselling program (a community-based, domestic violence-sexual assault service agency), and it appears that it was also successful in that regard as only 22% of the patients stated that they were unlikely to attend counselling. |
| Darnell 2015 | Harborview Medical Centre, Seattle, Washington, US | SANE-SART |  | Yes | FME conducted in the ED. ED social workers schedule victims for a follow-up medical/counselling appointment at Harborview Center for Sexual Assault and Traumatic Stress (HCSATS) to be held within two weeks of the ED visit. At the follow-up appointment, victims see the SANE coordinator, who herself occasionally provides services to rape victims in the ED, and a HCSATS (non-ED) social worker. The HCSATS provides evidence-based cognitive-behavioural therapy to Harborview patients and community members affected by rape and other traumatic events. All victims receive SANE and follow-up appointment services for free. Those who report the rape to police and pursue state-funded crime victims’ compensation may also obtain mental health services related to the assault at no cost. |
| Dhairyawan 2017 | 19 unspecified SARCs, UK | SARC (UK) | Yes |  | (19/21) had links with local Emergency Departments, GPs, social services and mental health services. |
| Du Mont 2004 | 15 SA/DVCC, Ontario, Canada | SA/DVCC | Yes |  | Little mention from professionals > The on-call sexual assault physician provides a number of services, in conjunction with the on-call nurse. They crisis counsel and offer emotional support to the victim, explain available treatment and forensic options to her/him, and provide appropriate referrals to support services operating within the community. They also offer comprehensive medical care including a complete physical examination. |
| Du Mont 2014 | 30 SA/DVTCs, Ontario, Canada | SA/DVTC | Yes |  | On-site follow-up care and counselling, and referral to community agencies for ongoing support. |
| Durmaz 2014 | Glade SARC, Worcester, England | SARC (UK) | Yes |  | Risk assessment for self-harm or any safeguarding concerns. Following the examination, the SARC team will discuss the type of support most appropriate for the victim. This may involve counselling or more practical support from an independent sexual violence adviser, or follow up with sexual health services or mental health services. |
| Edinburgh 2008 | Child Advocacy Centre, US | CAC | Yes |  | Referral for counselling |
| Fong 2016 | Philadelphia, Children’s Alliance Child Advocacy, US | CAC |  | Yes | Staff refer all children with a disclosure of CSA to one of six preferred mental health sites in Philadelphia that provide trauma-focused cognitive behavioural therapy. Specific referrals are made to reduce geographic and transportation barriers to services. Caregivers are typically asked to schedule appointments themselves, but Philadelphia Children’s Alliance staff will provide referral information directly to mental health sites to facilitate the linkage process. Philadelphia Children’s Alliance staff will then follow up with mental health sites and/or caregivers to ensure that children have successfully linked to services and offer additional assistance as needed. |
| Gibney 2014 | Lancashire Sexual Assault Forensic Examination (SAFE) centre, England | SARC (UK) | Yes |  | Complainants are seen and examined at the centre, and appropriate follow up arranged; be it with Genito-Urinary Medicine, Paediatrician referral, Social Services or via General Practitioner correspondence. Two dedicated Independent Sexual Violence Advocates (ISVA) follow up those seen at the centre, should they wish to access their support. One of these ISVAs is specifically for adults; the other is a Children's and Young Person's Advocate (CYPA), seeing children and their parents/carers, offering emotional and practical support as well as advising parents/carers of the process after attending the centre and of the criminal proceedings. They do not offer counselling but signpost or refer on to other services if psychological therapy or counselling is requested. The follow up for psychological support however, is less clear cut and the case below highlights how difficult organising psychological support can be. |
| Goddard 2015 | The Havens, London, England | SARC (UK) | Yes |  | Following FME, the Havens hand the care of children under 13 over to local paediatric and social care teams. The paediatricians surveyed report significant issues with the referral process and this should be improved as a matter of urgency. The Havens receive no information on the children they have referred or feedback on the outcomes from social care. The Havens do not receive feedback on their forensic results and rarely on case outcomes through the criminal justice system. |
| Hanson 1985 | Child and Adolescent  Sexual Abuse Resource  Centre (CASARC), San Francisco, US | CAC | Yes |  | Children who require longer term psychotherapy, usually the victims of incest or longstanding molestation, are referred to appropriate agencies. Referral criteria onward for psychological support not detailed. |
| Heke 2009 | The Havens, London, England | SARC (UK) | Yes |  | The paper was about an audit of risk identification tools and management pathways developed for the Havens. The identification tool comprised of a risk identification form completed at first contact with the service and at follow-up. Risk management included referral to A&E, Internal referral for clinical psychology or external referral to other services. |
| Herbert 2018 | 362 CACs, US | CAC | Yes |  | Referral to on or off-site MH service or counselling    45% of CACs provided mental health services only on site; 36% provided MH services only off-site; 13% provided services on and off-site; 5% did not provide a MH service as part of the CAC |
| Hicks 2017 | Midwest SANE program, US | SANE |  | Yes | Referred on to either counselling or crisis intervention by victim advocate  Advocates assumed the role of contacting patients post examination to inquire about their well-being and needs. The SANE program followed up only 28% of adult patients in 2015. |
| Holton 2018 | George Regional Hospital Thuthuzela Care Centre, Eden District, South Africa | TCC | Yes |  | George Hospital: During office hours, psychosocial support was provided by Thuthuzela staff. After hours, counselling was covered by community partners. Health care services were provided by the hospital’s emergency centre doctors within the Thuthuzela examination room. All follow-up consultations were provided by the hospital’s occupational health and safety professional nurse. |
| Kerr 2003 | The Haven, London, England | SARC (UK) | Yes |  | The follow-up clinic offers psychosocial support and short-term counselling at the Haven. Clients with needs that cannot be provided within the service are referred on, either locally or in their district of residence. Some clients will only wish to access counselling months or years later, and so written information about potential later sources of help is also provided. |
| Lovett 2004 | (1)St Mary’s SARC, Manchester, England | SARC (UK) |  | Yes | Immediate crisis support is provided by the crisis worker. At the end of the examination a debriefing session takes place with the crisis worker, where additional St Mary's services are outlined. Since the appointment of the Support Worker all attendees are re - contacted the next working day; she offers both to attend the statement-taking (if it has not already been done) and to facilitate access both to St Mary ’s services and other external organisations. Proactive follow-up is also part of the support worker’s role. Counselling is available, and there is no limit set on the number of sessions. St Mary’s, also, operates an open advice, information and referral service through the telephone, including an ‘out-of-hours’ service. |
|  | (2)REACH, Ellis Fraser Centre, Sunderland, England | SARC (UK) | Yes |  | Procedures are similar to those for St Mary’s, with the exception that the role of the Crisis Worker has to be fulfilled by a combination of a specialist police officer and the forensic doctor. Counselling services at REACH are limited to a set number of ten sessions, although t h e re is some flexibility in individual cases. REACH also offers group counselling in some cases, and is the only SARC to do so currently. |
|  | (3)REACH, Rhona Cross Centre, Newcastle, England | SARC (UK) | Yes |  | Same as REACH 1 |
|  | (4)STAR, West Yorkshire, England | SARC (UK) | Yes |  | Its primary role, therefore, has been to co-ordinate and commission local support and counselling for adults, and also offer immediate support and advice through a helpline. Counselling is commissioned from accredited counsellors, who are responsible for making arrangements with service users about the location and timing of sessions. The rationale here is that this enables access close to where service users live. A maximum of ten sessions will be paid for by STAR for any service user. Two innovative elements of the STAR model are Initial Support Workers and the Case Tracker. Initial Support Workers are volunteers, trained by STAR, who offer immediate crisis support for six weeks – this may be practical, access to information, as well as just someone to talk to. The preferred model is for service users to move from this more informal support into counselling. |
| Mathews 2013 | Two dedicated sexual assault centres in Cape Town, Western Province, South Africa | Other | Yes |  | Although all the children were referred for support services, only 12 of the 31 children accessed and received counselling, with the longest attendance being 10 weeks and the shortest being one visit. Counselling was offered by two organizations, but one saw children only 14 years and older. |
| Minden 1989 | Victim Care Service at the Boston City Hospital, US | Other | Yes |  | The service offers up to 4 supportive counselling sessions provided by a team of Masters-prepared psychiatric nurses and social workers. These clinicians act as advocates for the most vulnerable patients and make sure they are hooked up with the necessary other supports and services. Ongoing therapy operates on a referral basis as BCH doesn’t have the capacity to provide outpatient mental health services. The VCS maintains a linkage system with other community programs who offer individual, family and group therapy in order to provide continuity of care for its patients. |
| Musgrave 2014 | The Ferns centre in Suffolk and the Harbour Centre in Norfolk, SARCs, England | SARC (UK) | Yes |  | Refer on to MH services |
| Nesvold 2008 | Sexual Assault Centre, Oslo, Norway | SAC (Nordic countries) | Yes |  | 138/354 referred for psychotherapy |
| Rheingold 2013 | Child Advocacy Centre, South East region, US | CAC | Yes |  | Rape crisis advocate offered to support child and family through the medical examination process. Signposting by rape crisis advocate. |
| Robinson 2009 | Ynys SAFF, Cardiff, Wales | SARC (UK) | Yes |  | ISAVs signposted and referred clients to external counselling services, drug and alcohol services, mental health services |
| Loko Roka 2014 | Sans Frontieres sexual violence programmes, Masini and Niangara, Democtratic  Republic of Congo | Other | Yes |  | Referred to hospital for psychological care and provided systemic follow-up appointments were made for psychological care and for specific circumstances such as pregnancy. |
| Sacks 2008 | The Haven Whitechapel, London, England | SARC (UK) |  | Yes | SARC clients "are screened using a Risk Assessment Proforma to identify mental health issues" and "Data were collected from the forensic notes and follow-up notes". The service provides in-house mental health support (counselling or clinical psychology), but also refers to adolescent mental health services with active follow-up. |
| Schonbucher 2009 | Archway Glasgow SARC, Scotland | SARC (UK) | Yes |  | Follow-up services consist of support work and counselling. The support work comprises of advocacy and practical support alongside providing emotional support and accompaniment. The support worker refers people to other follow up services, including counselling. Counselling is envisaged as providing professional support to help the service users cope with the emotional and social consequences, with the aim off minimising negative repercussions on the service users’ mental health. Both counselling and support worker sessions take place in the SARC. |
| Smith 2006 | 10 unspecified CAC, US | CAC | Yes |  | Mental health referrals |
| Speck 2005 | (1)Government SANE program, Freehold, New Jersey  (2)Commercial SANE program, Los Angeles/ Orange County, California | SANE-SART |  | Yes | (1&2)They do counselling and mental health referrals. While the not-for-profit SANE program in Georgia does not follow up with the patients to determined health seeking behaviour, both the commercial and the government SANE program administrators do follow up. |
|  | (3)Non-profit SANE program, Athens, Georgia | SANE-SART | Yes |  | (3)They do counselling and mental health referrals. While the not-for-profit SANE program in Georgia does not follow up with the patients to determined health seeking behaviour, both the commercial and the government SANE program administrators do follow up… |
| Ward 2006 | “Rainbo Centres” Freetown, Kailahun, Kenema and Kono districts in Sierra Leone | Other) | Yes |  | Psychosocial counselling, referral and advocacy services. |
| Winters 2011 | SAFE Place Merseyside (SPM), Liverpool, England | SARC (UK) | Yes |  | On arrival at the Centre, clients will be able to access emotional and practical support from a Crisis Worker and will also be given information about how to access further care/counselling and support/advice on various criminal justice options. |
| Zijlstra 2017 | Centre for Sexual and Family Violence (CSFV), Nijmegen, Netehrlands | Other | Yes |  | A case manager, based at the academic Primary Health Care Centre, calls victims the day after acute care has been provided. This case manager is in charge of psychosocial follow-up care such as providing psychoeducation and referral to psychosocial or legal help, if necessary. Follow-up care can be done by phone or in face-to-face contacts, depending on the victims' preferences. The case manager screens for PTSD at one month and three months after the incident according to the NICE guidelines. The case manager keeps files, registering the victims' needs and action plans. The service has agreements with a centre for psychologists specialized in trauma. Victims are referred to this centre within two weeks if necessary. If victims do not want or need follow-up care, the case manager asks for the reason why and registers this as well. After three months, the case manager usually transfers the care to the victims' GP. |

**References**

Abrahams N, Jewkes R. Barriers to post exposure prophylaxis (PEP) completion after rape: a South African qualitative study. Cult Health Sex. 2010;12(5):471-84. Epub 2010/02/20. doi: 10.1080/13691050903556316. PubMed PMID: 20169481.

Ahrens C, Campbell R, Wasco S, Aponte G, Grubstein L, Davidson II W. Sexual Assault Nurse Examiner (SANE) programs: Alternative systems for service delivery for sexual assault victims. Journal of Interpersonal Violence. 2000;15(9):921-43. doi: 10.1177/088626000015009002.

Beauchamp BT. The development and suggested implementation of a program evaluation for a crisis intervention sexual abuse service program for children and families [Dissertation]. Lousville, Kenucky: Spalding University; 2000.

Bechtel K, Ryan E, Gallagher D. Impact of Sexual Assault Nurse Examiners on the Evaluation of Sexual Assault in a Pediatric Emergency Department. Pediatric Emergency Care. 2008;24(7):442-7. doi: 10.1097/PEC.0b013e31817de11d.

Belew S. A program evaluation for the mental health department of the Chicago Children's Advocacy Center [Dissertation]. Illinois, United States: The Chicago School of Professional Psychology; 2012.

Bennett S, Plint AC, MacKay M. A survey of the 16 Canadian child and youth protection programs: A threadbare patchwork quilt. Paediatr Child Health. 2007;12(3):205-9.

Bicanic I, Snetselaar H, De Jongh A, Van de Putte E. Victims' use of professional services in a Dutch sexual assault centre. European Journal of Psychotraumatology. 2014;5. doi: 10.3402/ejpt.v5.23645. PubMed PMID: 24959327.

Boykins ALD. Characteristics and follow-up of adult female victims of recent sexual assault [Dissertation]. Tennessee: The University of Tennessee Flealth Science Center; 2003.

Bramsen RH, Elklit A, Nielsen LH. A Danish Model for Treating Victims of Rape and Sexual Assault: The Multidisciplinary Public Approach. Journal of Aggression, Maltreatment & Trauma. 2009;18(8):886-905. doi: 10.1080/10926770903291811.

Brooker C, Durmaz E. Mental health, sexual violence and the work of Sexual Assault Referral centres (SARCs) in England. J Forensic Leg Med. 2015;31:47-51. Epub 2015/03/05. doi: 10.1016/j.jflm.2015.01.006. PubMed PMID: 25735784.

Brown R, Du Mont J, Macdonald S, Bainbridge D. A comparative analysis of victims of sexual assault with and without mental health histories: acute and follow-up care characteristics. J Forensic Nurs. 2013;9(2):76-83. Epub 2013/10/26. doi: 10.1097/JFN.0b013e31828106df. PubMed PMID: 24158128.

Campbell L, Keegan A, Cybulska B, Forster G. Prevalence of mental health problems and deliberate self-harm in complainants of sexual violence. Journal of Forensic and Legal Medicine. 2007;14(2):75-8. doi: 10.1016/j.jcfm.2006.02.026.

Campbell R, Patterson D, Adams AE, Diegel R, Coats S. A participatory evaluation project to measure SANE nursing practice and adult sexual assault patients' psychological well-being. J Forensic Nurs. 2008;4(1):19-28. Epub 2008/04/05. doi: 10.1111/j.1939-3938.2008.00003.x. PubMed PMID: 18387006.

Campbell R, Townsend SM, Long SM, Kinnison KE, Pulley EM, Adames SB, et al. Responding to sexual assault victims' medical and emotional needs: a national study of the services provided by SANE programs. Res Nurs Health. 2006;29(5):384-98. Epub 2006/09/16. doi: 10.1002/nur.20137. PubMed PMID: 16977639.

Chomba E, Murray L, Kautzman M, Haworth A, Kasese-Bota M, Kankasa C, et al. Integration of services for victims of child sexual abuse at the university teaching hospital one-stop centre. J Trop Med. 2010:7. Epub 2010/08/14. doi: 10.1155/2010/864760. PubMed PMID: 20706671; PubMed Central PMCID: PMCPMC2913632.

Ciancone AC, Wilson C, Collette R, Gerson LW. Sexual Assault Nurse Examiner programs in the United States. Annals of Emergency Medicine. 2000;35(4):353-7. PubMed PMID: 10736121.

Cooper R, Deller C. Caring for Adolescent Victims of Sexual Assault. Children Australia. 1982;7(1):16-20. doi: 10.1017/s0312897000011851.

Creighton CD, Jones AC. Psychological profiles of adult sexual assault victims. J Forensic Leg Med. 2012;19(1):35-9. Epub 2011/12/14. doi: 10.1016/j.jflm.2011.10.007. PubMed PMID: 22152446.

Darnell D, Peterson R, Berliner L, Stewart T, Russo J, Whiteside L, et al. Factors Associated With Follow-Up Attendance Among Rape Victims Seen in Acute Medical Care. Psychiatry. 2015;78(1):89-101. Epub 2015/07/15. doi: 10.1080/00332747.2015.1015901. PubMed PMID: 26168030; PubMed Central PMCID: PMCPMC4777603.

Deller CR, Fatin NA, Stewart LA. Sexual Assault Referral Centre, Perth: the first thirty months. Australian Family Physician. 1979;8(7):771-5. PubMed PMID: 485965.

Dhairyawan R, Shardlow K, Carroll M, Sacks R. A national survey of facilities for complainants of sexual assault. Int J STD AIDS. 2017;28(5):512-9. Epub 2016/07/13. doi: 10.1177/0956462416658413. PubMed PMID: 27402337.

Du Mont J, Macdonald S, White M, Turner L, White D, Kaplan S, et al. Client satisfaction with nursing-led sexual assault and domestic violence services in Ontario. J Forensic Nurs. 2014;10(3):122-34. Epub 2014/08/22. doi: 10.1097/JFN.0000000000000035. PubMed PMID: 25144583.

Du Mont J, Parnis D. The doctor's dilemma: caregiving and medicolegal evidence collection. Medicine & Law. 2004;23(3):515-29. PubMed PMID: 15532945.

Duddle M. Emotional sequelae of sexual assault. Journal of the Royal Society of Medicine. 1991;84:26-8. doi: 10.1177/014107689108400110.

Durmaz E. Rigorous attention ro detail. Nursing Standard. 2014;28(49):64-5.

Edinburgh L, Pape-Blabolil J, Harpin SB, Saewyc E. Assessing exploitation experiences of girls and boys seen at a Child Advocacy Center. Child Abuse Negl. 2015;46:47-59. Epub 2015/05/20. doi: 10.1016/j.chiabu.2015.04.016. PubMed PMID: 25982287; PubMed Central PMCID: PMCPMC4760762.

Edinburgh L, Saewyc E, Levitt C. Caring for young adolescent sexual abuse victims in a hospital-based children's advocacy center. Child Abuse Negl. 2008;32(12):1119-26. Epub 2008/12/02. doi: 10.1016/j.chiabu.2008.05.006. PubMed PMID: 19041133.

Elliott DM, Briere J. Forensic Sexual Abuse Evaluations of Older Children: Disclosures and Symptomatology. Behavioral Sciences and the Law. 1994;12:261-177.

Foley M. Rape: A feminist analysis of recent public service provisions for women with particular reference to the Sexual Assault Referral Centre [Dissertation]: University of Salford; 1992.

Fong HF, Bennett CE, Mondestin V, Scribano PV, Mollen C, Wood JN. Caregiver perceptions about mental health services after child sexual abuse. Child Abuse Negl. 2016;51:284-94. Epub 2015/11/26. doi: 10.1016/j.chiabu.2015.09.009. PubMed PMID: 26602155.

Gibney DR, Jones A. A crisis worker's observations on the psychosocial support for victims and families following child sexual abuse; a case study. J Forensic Leg Med. 2014;27:25-8. Epub 2014/10/08. doi: 10.1016/j.jflm.2014.07.001. PubMed PMID: 25287795.

Goddard A, Harewood E, Brennan L. Review of pathway following sexual assault for children and young people in London. NHS England, The Havens, Kings College Hospital London; 2015.

Goodyear F. Sexual Assault Centre, Auckland, New Zealand. Medicine & Law. 1989;8(3):297-302. PubMed PMID: 2516213.

Güney SA, Bağ Ö, Binic NC. An Overview of a Hospital-Based Child Advocacy Center's Experience from Turkey. J Child Sex Abus. 2018;27(5):476-89. Epub 2018/07/19. doi: 10.1080/10538712.2018.1483461. PubMed PMID: 30020034.

Hagemann CT, Helland A, Spigset O, Espnes KA, Ormstad K, Schei B. Ethanol and drug findings in women consulting a Sexual Assault Center--associations with clinical characteristics and suspicions of drug-facilitated sexual assault. J Forensic Leg Med. 2013;20(6):777-84. Epub 2013/08/06. doi: 10.1016/j.jflm.2013.05.005. PubMed PMID: 23910880.

Hanson G, Pascoe DJ. Intervention with sexually abused children. The Child and Adolescent Sexual Abuse Resource Center at San Francisco. Mobius. 1985;5(1):65-9. PubMed PMID: 10270438.

Heke S, Forster G, d'Ardenne P. Risk identification and management of adults following acute sexual assault. Sexual and Relationship Therapy. 2009;24(1):4-15. doi: 10.1080/14681990802641307.

Henry L, editor Hospital Care for Victims of Sexual Assault. National Conference on Rape Law Reform; 1980; Hobart, Tasmania: Australian Institute of Criminology.

Herbert JL, Walsh W, Bromfield L. A national survey of characteristics of child advocacy centers in the United States: Do the flagship models match those in broader practice? Child Abuse Negl. 2018;76:583-95. Epub 2017/10/11. doi: 10.1016/j.chiabu.2017.09.030. PubMed PMID: 28992959.

Hester M, Lilley SJ. More than support to court: Rape victims and specialist sexual violence services. Int Rev Vict. 2018;24(3):313-28. Epub 2018/08/17. doi: 10.1177/0269758017742717. PubMed PMID: 30111902; PubMed Central PMCID: PMCPMC6066863.

Hicks DL, Patterson D, Resko S. Lessons Learned From iCare: A Postexamination Text-Messaging-Based Program With Sexual Assault Patients. J Forensic Nurs. 2017;13(4):160-7. Epub 2017/11/28. doi: 10.1097/JFN.0000000000000175. PubMed PMID: 29176518.

Holton G, Joyner K, Mash R. Sexual assault survivors' perspectives on clinical follow-up in the Eden District, South Africa: A qualitative study. Afr J Prim Health Care Fam Med. 2018;10(1):e1-e7. Epub 2018/06/27. doi: 10.4102/phcfm.v10i1.1631. PubMed PMID: 29943600; PubMed Central PMCID: PMCPMC6018593.

Hubel GS, Campbell C, West T, Friedenberg S, Schreier A, Flood MF, et al. Child advocacy center based group treatment for child sexual abuse. J Child Sex Abus. 2014;23(3):304-25. Epub 2014/03/20. doi: 10.1080/10538712.2014.888121. PubMed PMID: 24641523.

Kerr E, Cottee C, Chowdhury R, Jawad R, Welch J. The Haven: a pilot referral centre in London for cases of serious sexual assault. BJOG: An International Journal of Obstetrics and Gynaecology. 2003;110(3):267-71. doi: 10.1046/j.1471-0528.2003.02233.x.

Kille ML. A sexual assault referral service based on a hospital in a small Australian town. Medical Journal of Australia. 1986;145(5):189-94.

Larsen ML, Hilden M. Male victims of sexual assault; 10 years' experience from a Danish Assault Center. J Forensic Leg Med. 2016;43:8-11. Epub 2016/07/09. doi: 10.1016/j.jflm.2016.06.007. PubMed PMID: 27391940.

Ledray LE. Sexual assault nurse clinician: an emerging area of nursing expertise. AWHONNS Clinical Issues in Perinatal & Womens Health Nursing 1993;4(2):180-90.

Lippert T, Favre T, Alexander C, Cross TP. Families who begin versus decline therapy for children who are sexually abused. Child Abuse Negl. 2008;32(9):859-68. Epub 2008/10/28. doi: 10.1016/j.chiabu.2008.02.005. PubMed PMID: 18950858.

Loko Roka J, Van den Bergh R, Au S, De Plecker E, Zachariah R, Manzi M, et al. One size fits all? Standardised provision of care for survivors of sexual violence in conflict and post-conflict areas in the Democratic Republic of Congo. PLoS One. 2014;9(10):e111096. Epub 2014/10/21. doi: 10.1371/journal.pone.0111096. PubMed PMID: 25329482; PubMed Central PMCID: PMCPMC4203825.

Lovett J, Regan L, Kelly L. Sexual Assault Referral Centres : developing good practice and maximising potentials. London: Home Office Research Study 285, 2004.

Lurie S, Boaz M, Golan A. Risk factors for rape re-victimisation: a retrospective analysis. J Obstet Gynaecol. 2013;33(8):865-7. Epub 2013/11/14. doi: 10.3109/01443615.2013.829031. PubMed PMID: 24219731.

Manning D, Majeed-Ariss R, Mattison M, White C. The high prevalence of pre-existing mental health complaints in clients attending Saint Mary's Sexual Assault Referral Centre: Implications for initial management and engagement with the Independent Sexual Violence Advisor service at the centre. J Forensic Leg Med. 2019;61:102-7. Epub 05 December 2018. doi: 10.1016/j.jflm.2018.12.001. PubMed PMID: 30551033.

Mathews S, Abrahams N, Jewkes R. Exploring mental health adjustment of children post sexual assault in South Africa. J Child Sex Abus. 2013;22(6):639-57. Epub 2013/08/09. doi: 10.1080/10538712.2013.811137. PubMed PMID: 23924175.

McLean IA, Balding V, White C. Further aspects of male-on-male rape and sexual assault in greater Manchester. Medicine, Science & the Law. 2005;45(3):225-32. PubMed PMID: 16117283.

Miller KE, Cranston CC, Davis JL, Newman E, Resnick H. Psychological Outcomes After a Sexual Assault Video Intervention: A Randomized Trial. J Forensic Nurs. 2015;11(3):129-36. Epub 2015/08/21. doi: 10.1097/JFN.0000000000000080. PubMed PMID: 26291847.

Minden P. The victim care service: A program for victims of sexual assault. Archive of Psychiatric Nursing. 1989;III(1):41-6.

Musgrave S, Pickup L, Maskrey V, Blyth A, Notley C, Holland R. A Health Needs Assessment for complainants of Sexual Assault in Norfolk and Suffolk UK: University of East Anglia, Norfolk Constanbulary, Suffold Constanbulary, 2014.

Nelson DD. An examination of predictors of sexual assault mental health treatment utilization in Asian American, Native Hawaiian, and Caucasian sexual assault survivors [Dissertation]. Hawaii: University of Hawaiʻi at Mānoa; 2016.

Nesvold H, Friis S, Ormstad K. Sexual assault centers: attendance rates, and differences between early and late presenting cases. Acta Obstet Gynecol Scand. 2008;87(7):707-15. Epub 2008/07/09. doi: 10.1080/00016340802189847. PubMed PMID: 18607821.

Nixon RDV, Best T, Wilksch SR, Angelakis S, Beatty LJ, Weber N. Cognitive Processing Therapy for the Treatment of Acute Stress Disorder Following Sexual Assault: A Randomised Effectiveness Study. Behaviour Change. 2016;33(4):232-50. doi: 10.1017/bec.2017.2.

Olsen A, Majeed-Ariss R, Teniola S, White C. Improving service responses for people with learning disabilities who have been sexually assaulted: An audit of forensic services. British Journal of Learning Disabilities. 2017;45(4):238-45. doi: 10.1111/bld.12200.

Pillai M, Paul S. Facilities for complainants of sexual assault throughout the United Kingdom. J Clin Forensic Med. 2006;13(4):164-71. Epub 2006/03/28. doi: 10.1016/j.jcfm.2006.02.010. PubMed PMID: 16564201.

Ranney ML, Rennert-May E, Spitzer R, Chitai MA, Mamlin SE, Mabeya H. A novel ED-based sexual assault centre in western Kenya: description of patients and analysis of treatment patterns. Emerg Med J. 2011;28(11):927-31. Epub 2010/10/16. doi: 10.1136/emj.2010.096412. PubMed PMID: 20947922.

Rheingold AA, Danielson CK, Davidson TM, Self-Brown S, Resnick H. Video Intervention for Child and Caregiver Distress Related to the Child Sexual Abuse Medical Examination: A Randomized Controlled Pilot Study. Journal of Child and Family Studies. 2013;22(3):386-97. doi: 10.1007/s10826-012-9591-3.

Robinson AL, Hudson K, Brookman F. A Process Evaluation of Ynys Saff, the Sexual Assault Referral Centre in Cardiff. Wales: University of Cardiff, 2009.

Ruch LO, Chandler S. An Evaluation of a Center for Sexual Assault Victims. Women & Health. 1980;5(1):45-64. doi: 10.1300/J013v05n01_05.

Sacks RJ, Cybulska BA, Forster GE. Referral of young people attending a sexual assault referral centre to mental health services. Int J STD AIDS. 2008;19(8):557-8. Epub 2008/07/30. doi: 10.1258/ijsa.2008.008050. PubMed PMID: 18663045.

Schei B, Sidenius K, Lundvall L, Ottesen GL. Adult victims of sexual assault: acute medical response and police reporting among women consulting a center for victims of sexual assault. Acta Obstetricia et Gynecologica Scandinavica. 2003;82(8):750-5. PubMed PMID: 12848647.

Schonbucher V, Kelly L, Horvath M. Archway: Evaluation of the pilot Scottish rape and sexual assault referral center. UK: London Metropolitan University, Child and Woman Abuse Studies Unit; 2009.

Sidenius K, Pedersen B. Prevention of victimization following sexual assaults. NORA - Nordic Journal of Feminist and Gender Research. 2004;12(1):48-57. doi: 10.1080/08038740410005767.

Signal T, Taylor N, Botros H, Prentice K, Lazarus K. Whispering to Horses: Childhood Sexual Abuse, Depression and the Efficacy of Equine Facilitated Therapy. Sexual Abuse in Australia and New Zealand. 2013;5(1):24-32.

Smith DW, Witte TH, Fricker-Elhai AE. Service outcomes in physical and sexual abuse cases: a comparison of child advocacy center-based and standard services. Child Maltreat. 2006;11(4):354-60. Epub 2006/10/18. doi: 10.1177/1077559506292277. PubMed PMID: 17043320.

Speck PM. Program evaluation of current SANE services to victim populations in three cities [Dissertation]. Tennessee, US: University of Tennessee Health Science Center; 2005.

Vik BF, Nottestad JA, Schei B, Rasmussen K, Hagemann CT. Psychosocial Vulnerability Among Patients Contacting a Norwegian Sexual Assault Center. J Interpers Violence. 2016;34(10):2138-57. Epub 2016/07/28. doi: 10.1177/0886260516659657. PubMed PMID: 27449896.

Ward J, Marsh M. Sexual violence against women and girls in war and its aftermath: Realities, responses and required resources. Symposium on Sexual Violence in Conflict and Beyond2006. p. 1-34.

Winters L. Sexual Violence Needs Assessment For Merseyside. UK: Liverpool Public Health Observatory, 2011 Aug. Observatory Report Series No. 85.

Zijlstra E, Esselink G, Moors ML, LoFoWong S, Hutschemaekers G, Lagro-Janssen A. Vulnerability and revictimization: Victim characteristics in a Dutch assault center. J Forensic Leg Med. 2017;52:199-207. Epub 2017/09/30. doi: 10.1016/j.jflm.2017.08.003. PubMed PMID: 28961551.

Zilkens RR, Smith DA, Mukhtar SA, Semmens JB, Phillips MA, Kelly MC. Male sexual assault: Physical injury and vulnerability in 103 presentations. J Forensic Leg Med. 2018;58:145-51. Epub 2018/07/08. doi: 10.1016/j.jflm.2018.05.009. PubMed PMID: 29981506.
