# Supplementary material for: The identification and treatment of mental health and substance misuse problems in sexual assault services: A systematic review
Source: PLoS One. 2020 Apr 10;15(4):e0231260. doi: 10.1371/journal.pone.0231260 (PMC7147790; doi:10.1371/journal.pone.0231260)
Supplement: S5 File — (DOCX) [file pone.0231260.s005.docx]

**Table 5. Stakeholders’ views – General recommendations relevant to mental health and substance use**

| **Recommendation** | **Studies (n)** | **Stakeholder group(s)** |
| --- | --- | --- |
| **Access and referrals to sexual assault services** |  |  |
| Sexual assault services should be accessible 24/7 | 5 | Service users (Du Mont 2014)  Staff (Belew 2011)  Other professional stakeholders (Robinson 2009)  Mixed stakeholders (Cole 2007, COSAI 2012) |
| Support from sexual assault services should be clearly advertised as being available for LGBT people | 1 | Mixed stakeholders (Harvey 2014) |
| Self-referrals to sexual assault services should be possible | 1 | Mixed stakeholders (COSAI 2012) |
| Phone lines in sexual assault services should be answered promptly | 1 | Service users (Du Mont 2014) |
| Sexual assault services should be free for service users | 1 | Staff (Ahrens 2000) |
| Video-conferencing can be used to allow clinicians in sexual assault services to support and advise GPs in assessing people locally following a sexual assault – eg in rural areas where access to a sexual assault service is challenging. | 1 | Other stakeholders (Burton 2002) |
| Sexual assault services should be flexible about timing and location of appointments (e.g. offering after –school counselling for children service users) | 3 | Staff (Belew 2011)  Mixed stakeholders (COSAI 2012)  Mixed stakeholders (Harvey 2014) |
| should address language barriers and offer a multi-lingual service | 2 | Staff (Belew 2011)  Mixed stakeholders (COSAI 2012) |
| Havens should be able to examine historic cases of CSA | 1 | Mixed stakeholders (Goddard 2015) |
| **Treatment and support in sexual assault services** |  |  |
| Sexual assault services should offer a social model of care that addresses the social and emotional needs of service users to the same degree as their medical and evidential needs | 1 | Staff (Schönbucher 2009) |
| All support staff in sexual assault services should receive ongoing training on counselling and support for victims of sexual assault | 1 | Staff (Schönbucher 2009) |
| Sexual assault services should offer a “one-stop shop” providing multi-agency holistic support to meet service users’ needs following an assault | 11 | Mixed stakeholders (Campbell 1998,Lovett 2004, COSAI 2012, Musgrave 2014, Robinson 2011, Ruch 1980) Staff (Clark 1998, Maier 2012, Cowley 2014)  Service users (Ericksen 2002)  Other professional stakeholders (Robinson 2009) |
| Sexual assault services should offer “comfort care” (e.g. food, showers, clean clothes) | 3 | Staff (Cowley 2014)  Service users (Ericksen 2002)  Mixed stakeholders (Ruch 1980) |
| Sexual assault services should assess service users’ capacity to consent to all treatments, procedures or assessments | 3 | Service users (Campbell 2013)  Staff (Cowley 2014) |
| Sexual assault services should help service users access safe housing when required | 1 | Mixed stakeholders (Campbell 1998) |
| Specialist support within sexual assault services is needed for trans-gender and trans-sexual clients | 1 | Mixed stakeholders (Musgrave 2014) |
| Staff in sexual assault services should be skilled in LGBT people’s needs and experiences | 1 | Mixed stakeholders (Harvey 2014) |
| **Continuity of care in sexual assault services** |  |  |
| Sexual assault services should work with referrers to minimise service users having to repeat their story on multiple occasions (e.g. by police bringing the person promptly to the sexual assault service to take an initial statement there) | 2 | Other professional stakeholders (Robinson 2009)  Mixed stakeholders (Ruch 1980) |
| Staff in sexual assault services must ensure service users know they can decline a forensic examination/assessment and still receive healthcare and other support | 3 | Staff (Ahrens 2000)  Mixed stakeholders (Ruch 1980)  Service users (Du Mont 2009) |
| Collaboration is needed between sexual assault services and Age-related organisations to facilitate support for older survivors of sexual assault | 1 | Mixed stakeholders (Bows 2018) |
| Provision of a schedules appointment is needed to enable access to follow-up care | 1 | Service users (Holton 2018) |
| An individually tailored care plan and provision of a familiar trusted healthcare provider is needed to improve accessibility to follow-up care | 1 | Service users (Holton 2018) |
| **Sexual assault services - users’ experience** |  |  |
| Staff in sexual assault services should treat service users with kindness and compassion | 4 | Staff (Campbell 2006)  Service users (Du Mont 2014)  Other professional stakeholders (Robinson 2009)  Mixed stakeholders (Lovett 2004)  Staff (Schönbucher 2009) |
| Staff in sexual assault services should communicate to service users that they are believed about their assault | 6 | Service users (Campbell 2013, Ericksen 2002)  Staff (Campbell 2005, Maier 2012)  Other professional stakeholders (Robinson 2009)  Mixed stakeholders (Lovett 2004) |
| Service users should have clear information, choice and control about what happens to them at the sexual assault service at all times | 6 | Staff (Ahrens 2000, Campbell 2006)  Mixed stakeholders (Campbell 1998, Lovett 2004)  Service users (Campbell 2013)  Service users (Schönbucher 2009) |
| Support to female service users should always be available from female staff | 5 | Staff (Ahrens 2000)  Service users (Ericksen 2002)  Mixed stakeholders (Lovett 2004, COSAI 2012)  Staff (Schönbucher 2009) |
| Two staff should be present during examinations and evidence gathering (one to talk to and reassure the service user) | 3 | Staff (Downing 2012, Maier 2012)  Mixed stakeholders (Lovett 2004) |
| Use of touch by staff (e.g. a hug, hand-holding) can be comforting when used sensitively. | 1 | Service users (Ericksen 2002) |
| Use by staff of everyday chat and humour, and breaks from procedures, can help service users contain distress | 1 | Service users (Campbell 2013) |
| Assessments and procedures i should not be rushed | 3 | Staff (Campbell 2006)  Mixed stakeholders (Campbell 1998)  Service users (Campbell 2013) |
| Service users prefer minimal, but reassuring conversation with staff during initial consultation | 1 | Service users (Holton 2018) |
| Service users welcome accessible information and a proactive contact approach by staff in sexual assault services | 1 | Service users (Schönbucher 2009) |
| **Sexual assault services - Organisation** |  |  |
| Sexual assault services are required for children and adults | 1 | Staff (Robinson 2011) |
| Sexual assault services should be located separately from hospital emergency departments, health or police premises (to maximise privacy and their sole role to support people following sexual assault) | 3 | Service users (Du Mont 2014)  Staff (Robinson 2011)  Mixed stakeholders (Cole 2007) |
| Sexual assault services should be included and considered in area-level crime policies and strategies | 1 | Staff (Robinson 2011) |
| Data from sexual assault services should be used to identify high risk groups or locations for sexual assault. These findings should be shared with police and other agencies. | 1 | Other professional stakeholders (Robinson 2009) |
| Sexual assault services should actively publicise their service and engage with other agencies, to encourage prompt referrals | 3 | Staff (Ahrens 2000)  Mixed stakeholders (Campbell 1998)  Service users (Du Mont 2014) |
