# Supplementary material for: The identification and treatment of mental health and substance misuse problems in sexual assault services: A systematic review
Source: PLoS One. 2020 Apr 10;15(4):e0231260. doi: 10.1371/journal.pone.0231260 (PMC7147790; doi:10.1371/journal.pone.0231260)
Supplement: S6 File — (PDF) [file pone.0231260.s006.pdf]

NHS
National Institute for Health Research

The identification and treatment of mental health and substance misuse problems in Sexual Assault Referral Centres (SARCs): a systematic review

Theodora Stefanidou, Brynmor Lloyd-Evans, Elizabeth Hughes, Charles Brooker, Amanda Edmonson, Gail Gilchrist, Kylee Trevillion

Citation

Theodora Stefanidou, Brynmor Lloyd-Evans, Elizabeth Hughes, Charles Brooker, Amanda Edmonson, Gail Gilchrist, Kylee Trevillion. The identification and treatment of mental health and substance misuse problems in Sexual Assault Referral Centres (SARCs): a systematic review. PROSPERO 2018 CRD42018119706 Available from:
http://www.crd.york.ac.uk/PROSPERO/display_record.php?ID=CRD42018119706

Review question

The study is a systematic review of previously reported studies and will require no ethical approval, or additional consent from participants. It aims to systematically review the comparative evidence for SARCs regarding mental health and/or substance misuse outcomes, addressing the following three research questions:

1) What are the approaches to the prevention, identification and/or treatment of mental health and/or substance misuse problems of different SARC service models identified in the literature?

2) What are the findings from quantitative evaluations of SARCs or equivalent services regarding mental health and/or substance misuse?

3) What are stakeholders' views and policy recommendations on how SARCs or equivalent services should identify, reduce risk of, and/or support people with mental health and/or substance misuse following a sexual assault?

Searches

We will search the following electronic databases: PsycINFO, MEDLINE, International Bibliography of the Social Sciences and CINAHL.

We will use broad search terms for SARCs and synonyms. The search will be limited to humans and no language restrictions will be imposed.

We will search for additional studies and grey literature, including conference proceedings, through Google Scholar and Zetoc databases.

We will search Primary Care Commissioning, Department of Health and NHS England websites for relevant policy guidance. We will, also, consult experts in the field to identify additional citations.

Types of study to be included

The following types of study published in peer-reviewed journals or grey literature will be included for each of our three research questions:

1) Literature including descriptive or evaluation studies, which specify SARC service models or equivalent services, including information about how mental health and substance misuse problems are identified, prevented and/or treated.

2) Quantitative comparison studies of any design which compare SARCs or equivalent services with standard care; which compare two different SARC service models; or which evaluate two different interventions/packages of care within a SARC service.

3) i. Qualitative interviews, focus groups or surveys of stakeholders' views which include any report of participants' views or recommendations regarding the prevention, identification and/or treatment of mental health and/or substance misuse problems in SARCs or equivalent services.

ii. Policy guidelines and service specifications from government or expert bodies regarding mental health and/or substance misuse problems identification, prevention and/or treatment in the SARC service model.

Condition or domain being studied

We will examine a broad range of evidence about how centres providing multi-agency, one-stop-shop provision for people who have experienced recent or non-recent sexual assault, can be implemented effectively to reduce risk, identify and/or address mental health and/or substance misuse problems. In England, such services are called Sexual Assault Referral Centres (SARCs).

A service will be considered as equivalent to SARC if it offers specialist support to people who have experienced sexual assault within a single service including:

a. Collection of forensic evidence and legal statements; and

b. Healthcare provision.

Participants/population

Inclusion criteria: adult and/or child stakeholders of SARCs or equivalent services.

Exclusion criteria: adult and/or child stakeholders of any other service except SARCs or equivalent services.

Intervention(s), exposure(s)

Inclusion criteria: SARCs or equivalent services, SARC service models or equivalent models, interventions/packages of care within a SARC or equivalent service.

Exclusion criteria: other sexual assault services not meeting our definition of SARCs or equivalent services.

Comparator(s)/control

Inclusion criteria: for quantitative comparison studies to be included in our research Q2, the comparator can be standard care, SARC service models or equivalent models, intervention/packages of care within a SARC or equivalent service.

Context

Inclusion criteria: SARCs or equivalent services.

Exclusion criteria: Sexual assault services that are not SARCs, or SARCs equivalent.

Main outcome(s)

Inclusion criteria: for quantitative comparison studies (research Q2), any measure of mental health, wellbeing or substance misuse at end of SARC treatment or longer-term follow-up; use of inpatient or other mental health crisis services at follow-up.

Exclusion criteria: for quantitative comparison studies (research Q2), forensic and physical health outcomes at the end of SARC treatment or longer-term follow-up.

Additional outcome(s)

Inclusion criteria: for quantitative comparison studies (research Q2), any measure of social functioning, quality of life or satisfaction with care at end of SARC treatment or longer-term follow-up.

Exclusion criteria: for quantitative comparison studies (research Q2), forensic and physical health outcomes at the end of SARC treatment or longer-term follow-up.

Data extraction (selection and coding)

All retrieved records will be screened by two independent reviewers. Any disagreements will be resolved by consensus, and when necessary after discussion with a third researcher. A data extraction schedule will be developed and used to retrieve information from included studies regarding: first author, year of publication, country, study design, number of participants, setting, SARC characteristics and any outcomes evaluated and results. For quantitative studies, the characteristics of the SARC services described in the study and (where applicable) of the comparison services will also be recorded. For qualitative studies, questionnaire surveys and government or other expert guidance, the main findings/recommendations regarding SARC service delivery or organisation in regards to mental health and/or substance misuse will be recorded.

Risk of bias (quality) assessment

Risk of bias will be assessed for all evaluation studies meeting the inclusion criteria for review question 2, and qualitative studies included for review question 3. It will be conducted using the Mixed Methods Appraisal Tool (MMAT) (Pace et al., 2012). This is an established assessment tool that it was chosen because of its application in assessing quantitative, qualitative and mixed methods primary studies. According to preliminary searches all three methodologies are likely to be represented in the papers included in the review. No quality assessment of policy guidelines or descriptive studies of SARC characteristics will be undertaken.

Strategy for data synthesis

A narrative synthesis will be undertaken, following ESRC guidelines (Popay J, 2005). The methods and findings from individual included studies will be summarized. For quantitative evaluation studies, the consistency of results between different studies will be considered, with consideration of the relevance of the evidence to child and adult participants, time since assault, and whether people have a pre-existing mental health condition and mental health service support or not. The nature of SARC service provision in quantitative evaluation studies will be described, with reference to SARC service models identified, and potential mechanisms of effect and key organizational/resource requirements in SARCs will be considered. Recommendations from policy guidelines for SARCs regarding mental health and substance misuse will be extracted and summarized. Relevant themes from qualitative papers will be summarized, with differences in perspective between or within stakeholder groups and recommendations for SARC service provision highlighted. The overall strength of the evidence regarding approaches to identification and treatment of mental health and substance misuse in SARCs will be considered, with reference to the quality of included studies and the consistency and scope of findings.

Analysis of subgroups or subsets

None planned.

Contact details for further information

Theodora Stefanidou
theodora.stefanidou.15@ucl.ac.uk

Organisational affiliation of the review

University College London (UCL)
https://www.ucl.ac.uk/

Review team members and their organisational affiliations

Ms Theodora Stefanidou. University College London (UCL)
Dr Brynmor Lloyd-Evans. University College London (UCL)
Professor Elizabeth Hughes. University of Huddersfield
Professor Charles Brooker. Royal Holloway, University of London
Dr Amanda Edmonson. University of Huddersfiled
Dr Gail Gilchrist. King's College London
Dr Kylee Trevillion. King's College London

Collaborators

Dr Steven Ariss. University of Sheffield
Professor Michael Lucock. University of Huddersfield
Ms Fay Maxted. The Survivors Trust

Dr Rebekah Shallcross. University of Bristol
Miss Rachael Hunter. University College London (UCL)
Dr Sarah Kendal. University of Huddersfield
Dr Karen Tocque. University of Huddersfield
Dr Christine Margaret Smith. University of Huddersfield

Anticipated or actual start date
01 August 2018

Anticipated completion date
31 May 2019

Funding sources/sponsors
This review presents independent research conducted as part of a study entitled “The effectiveness of Sexual Assault Referral Centres with regard to Mental Health and Substance Use: A National Mixed Method Study”, which is funded by the National Institute for Health Research (NIHR) (Reference Number: NIHR HSDR 16/117/03 HUGHES)

Conflicts of interest

Language
English

Country
England

Stage of review
Review_Ongoing

Subject index terms status
Subject indexing assigned by CRD

Subject index terms
Adult; Child; Drug Users; Humans; Mental Health; Mental Disorders; Primary Prevention; Referral and Consultation; Sex Offenses; Substance Abuse Detection; Substance-Related Disorders; Violence

Date of registration in PROSPERO
17 December 2018

Date of publication of this version
17 December 2018

Details of any existing review of the same topic by the same authors

Stage of review at time of this submission

**Stage**	**Started**	**Completed**
Preliminary searches	Yes	Yes
Piloting of the study selection process	Yes	Yes
Formal screening of search results against eligibility criteria	Yes	No
Data extraction	Yes	No
Risk of bias (quality) assessment	No	No
Data analysis	No	No

Versions

17 December 2018

PROSPERO
This information has been provided by the named contact for this review. CRD has accepted this information in good faith and registered the review in PROSPERO. CRD bears no responsibility or liability for the content of this registration record, any associated files or external websites.
